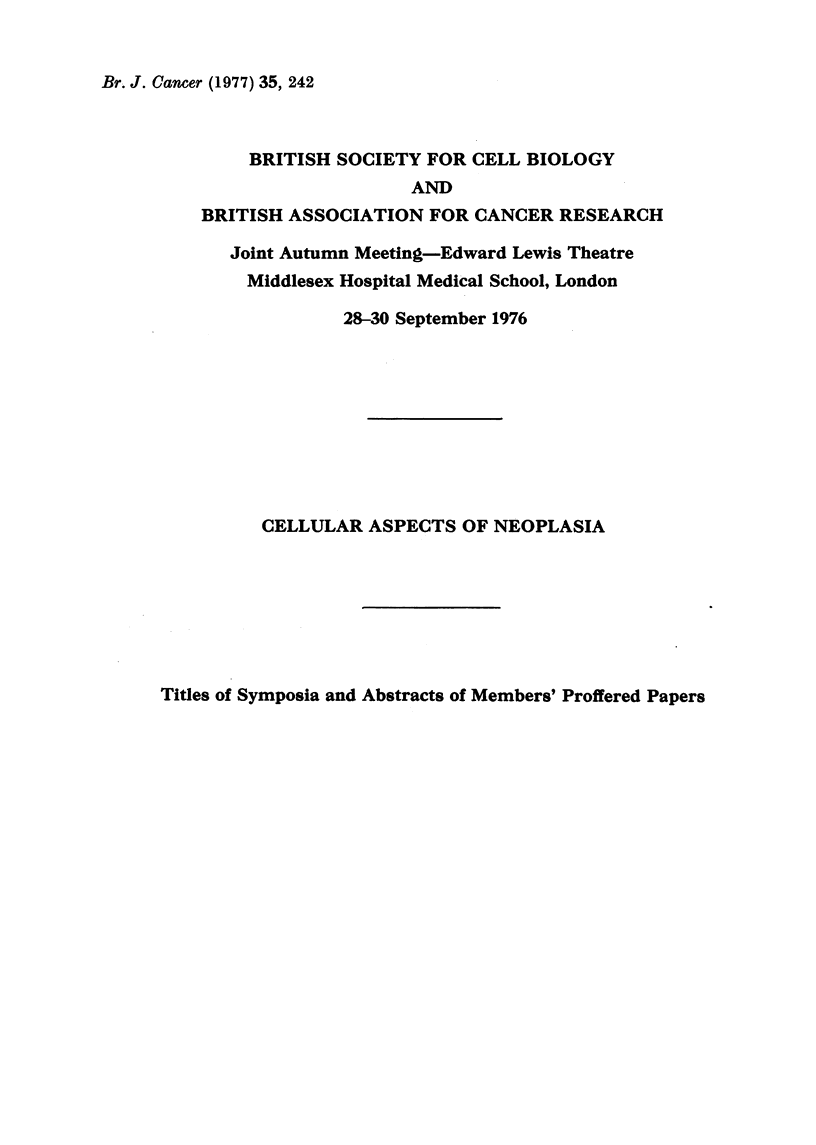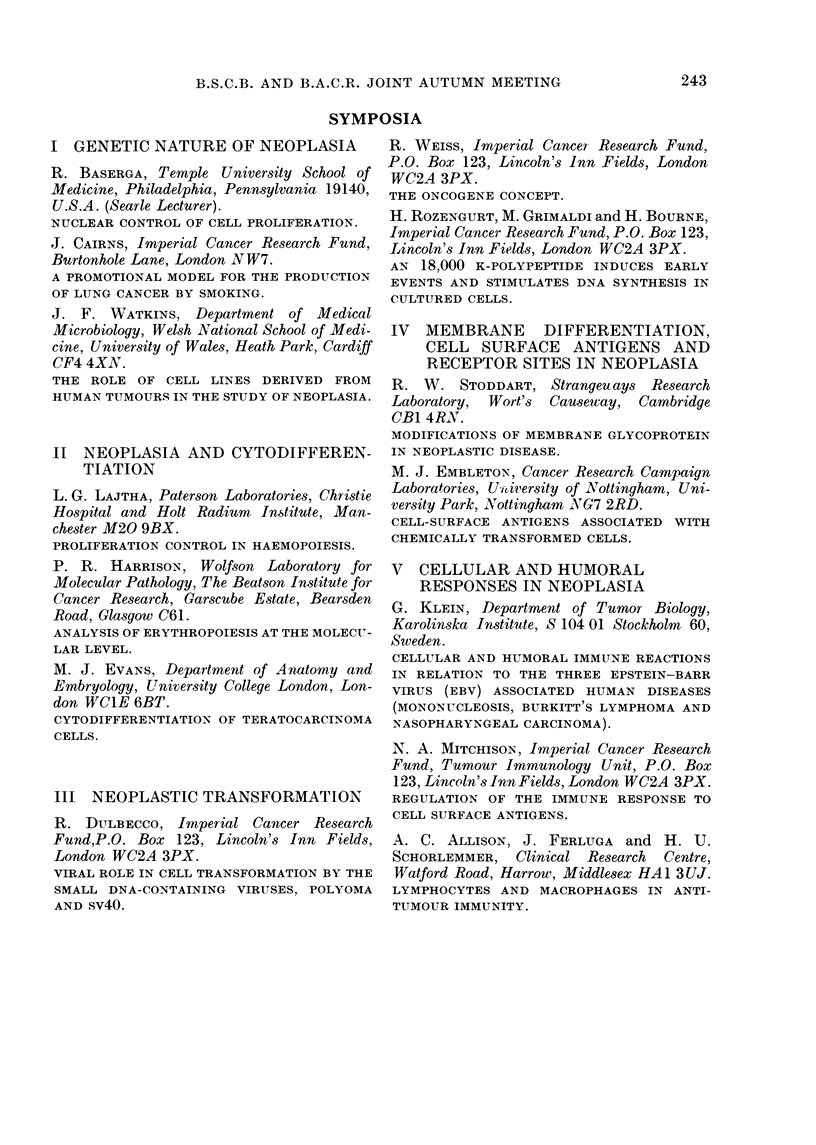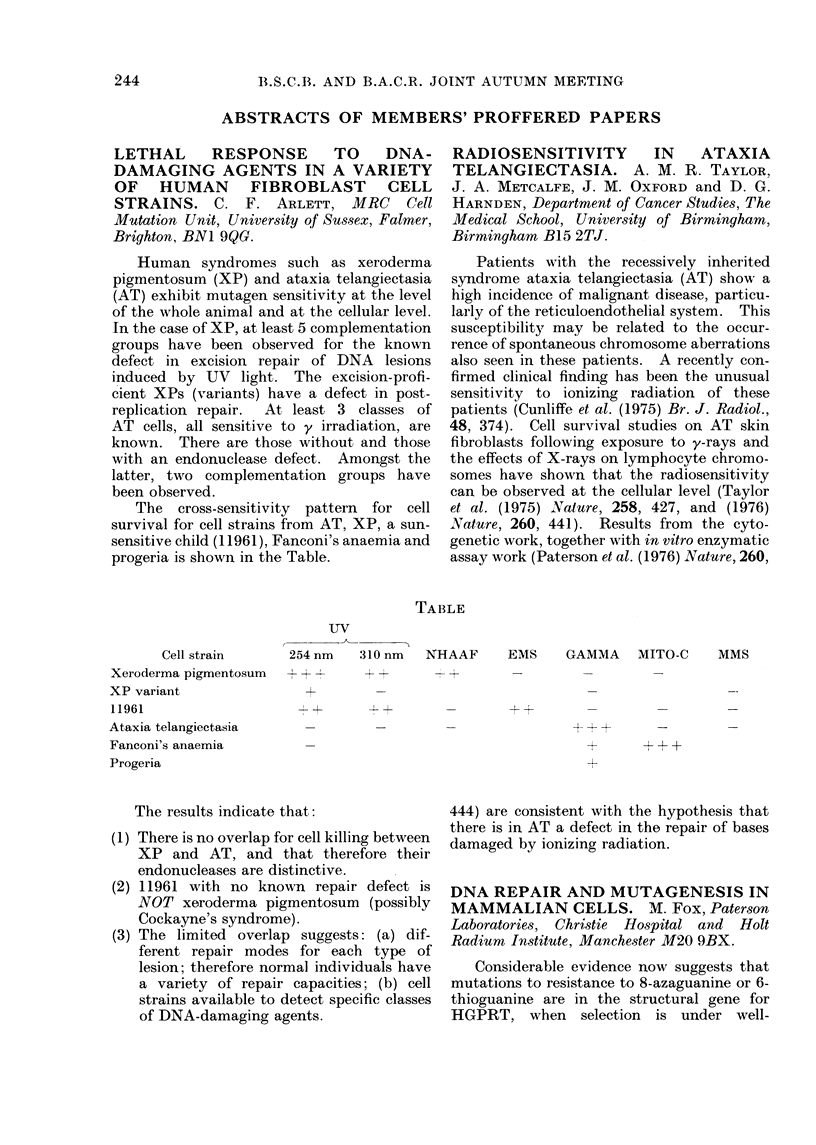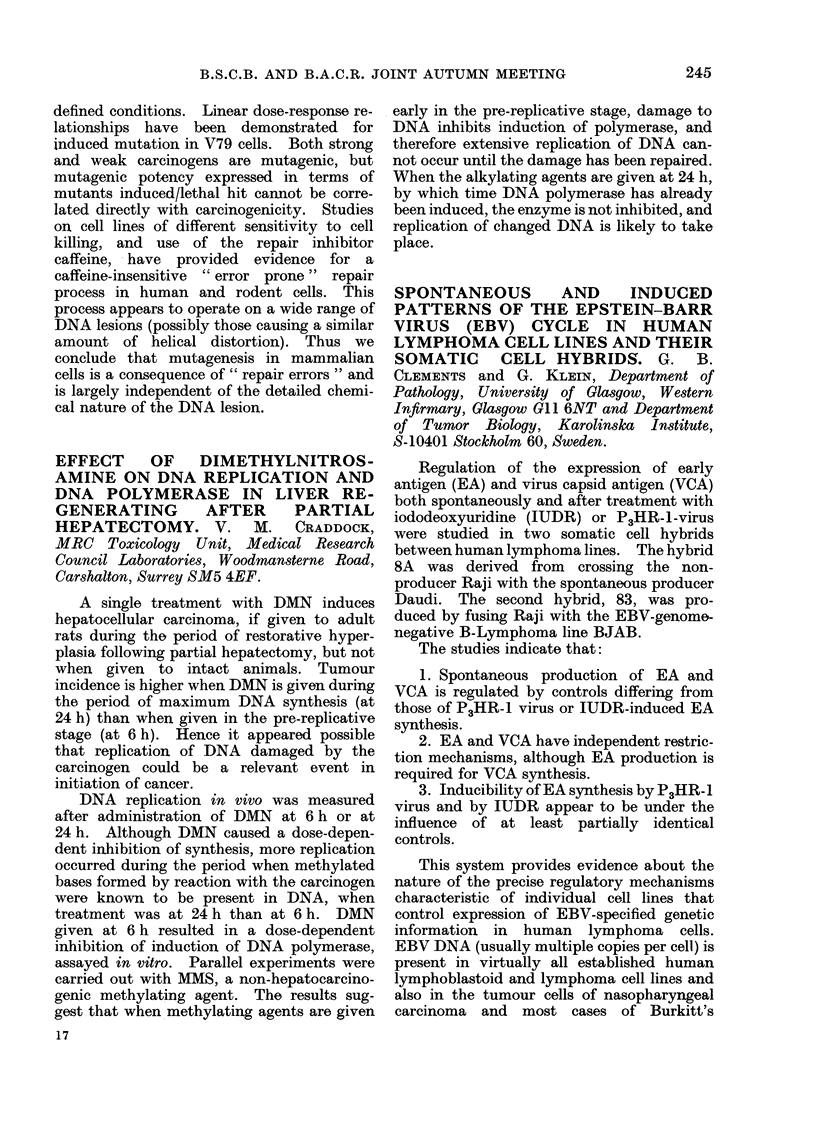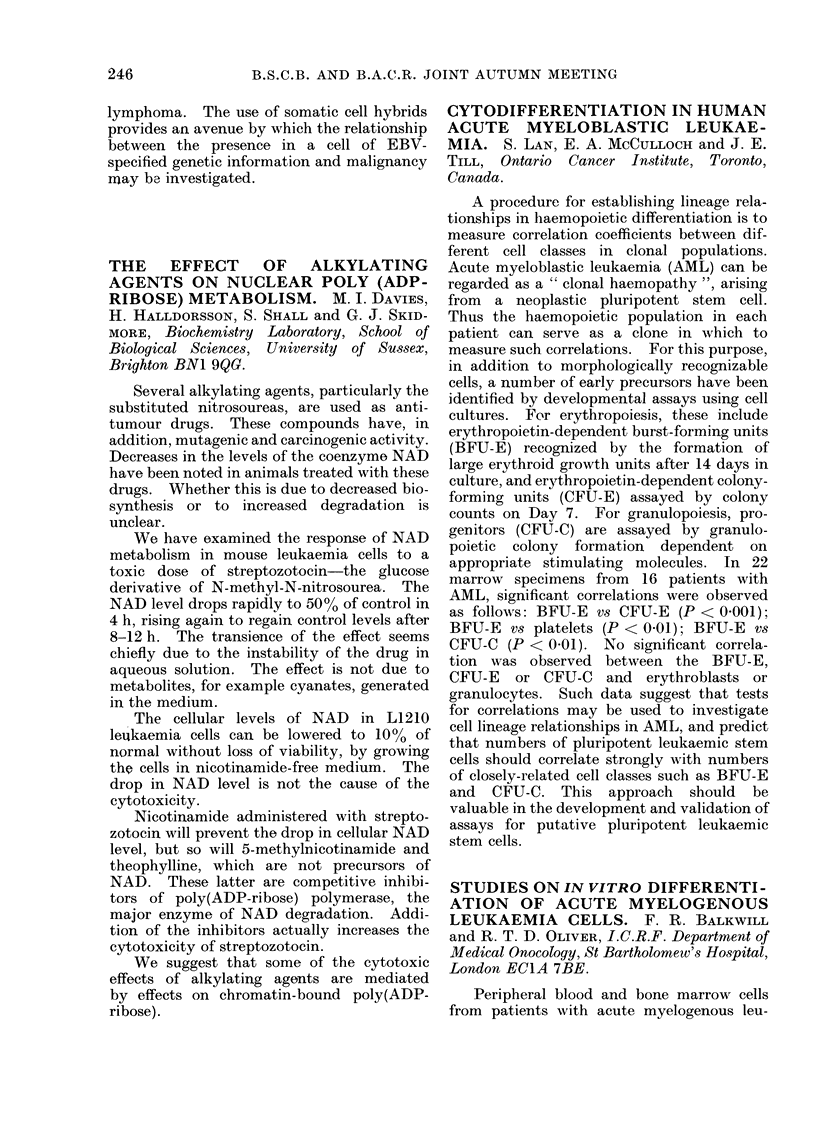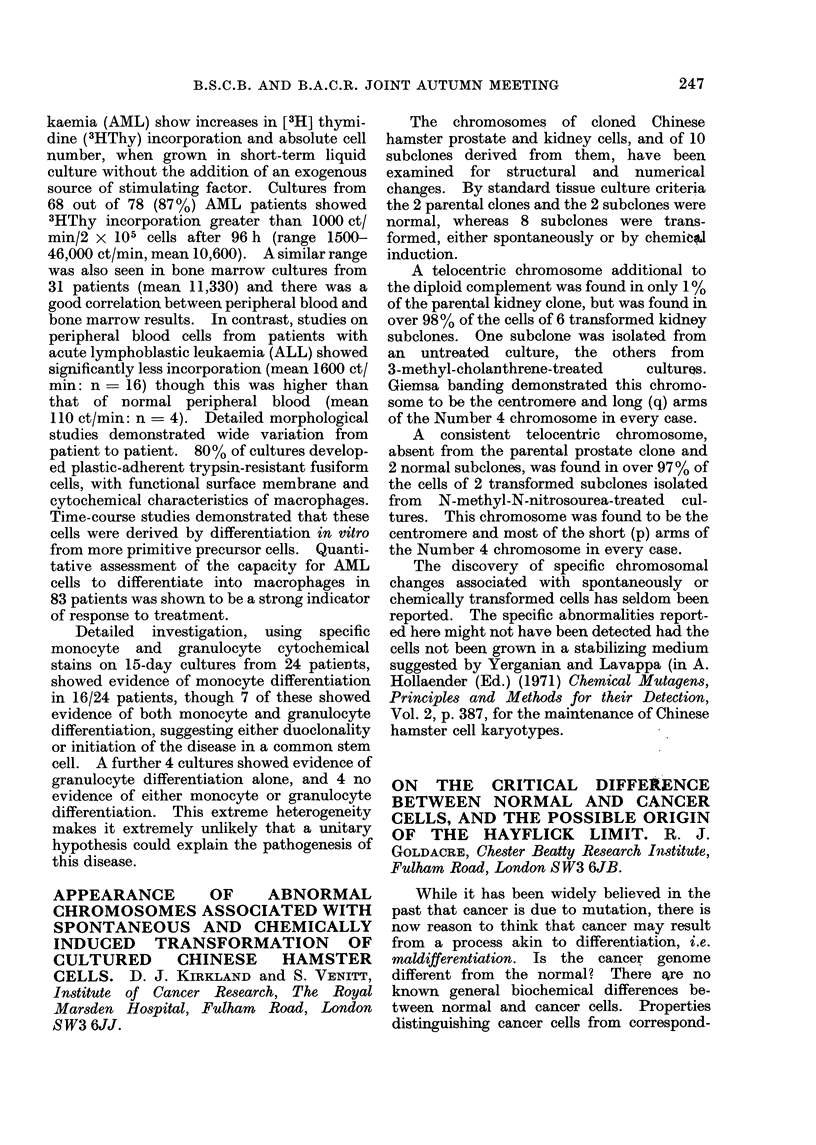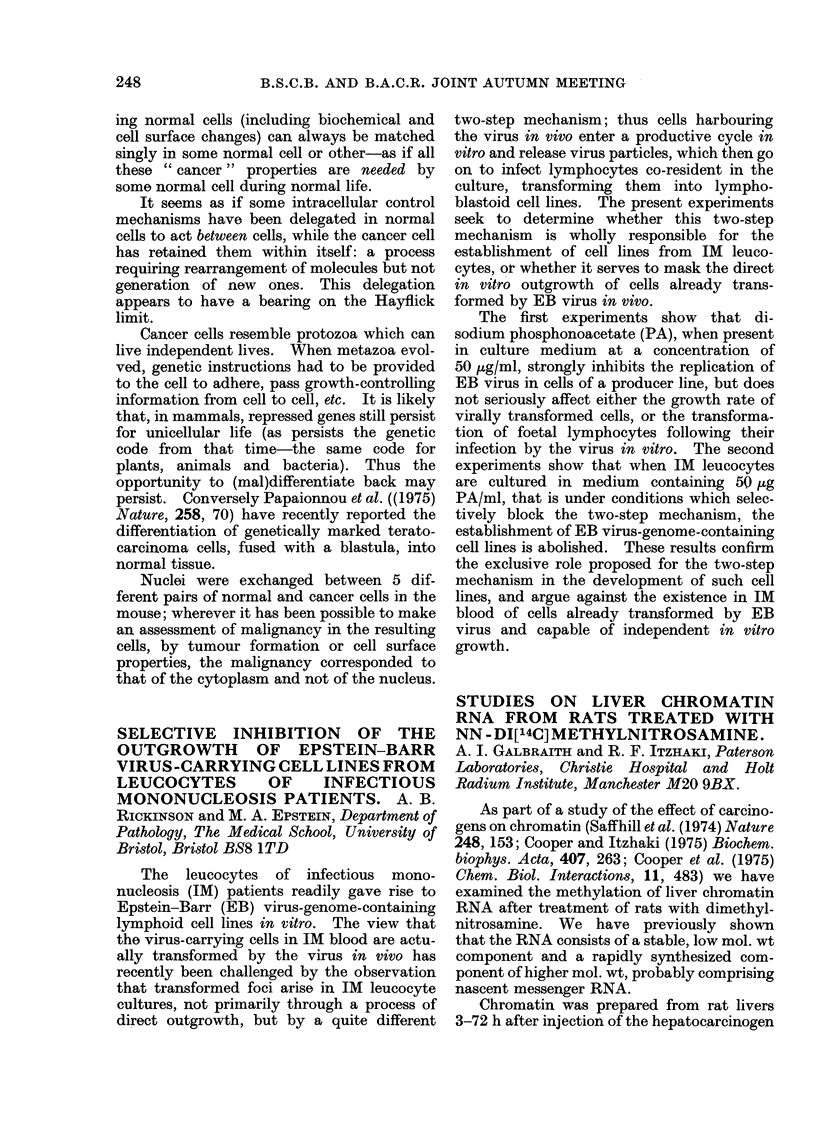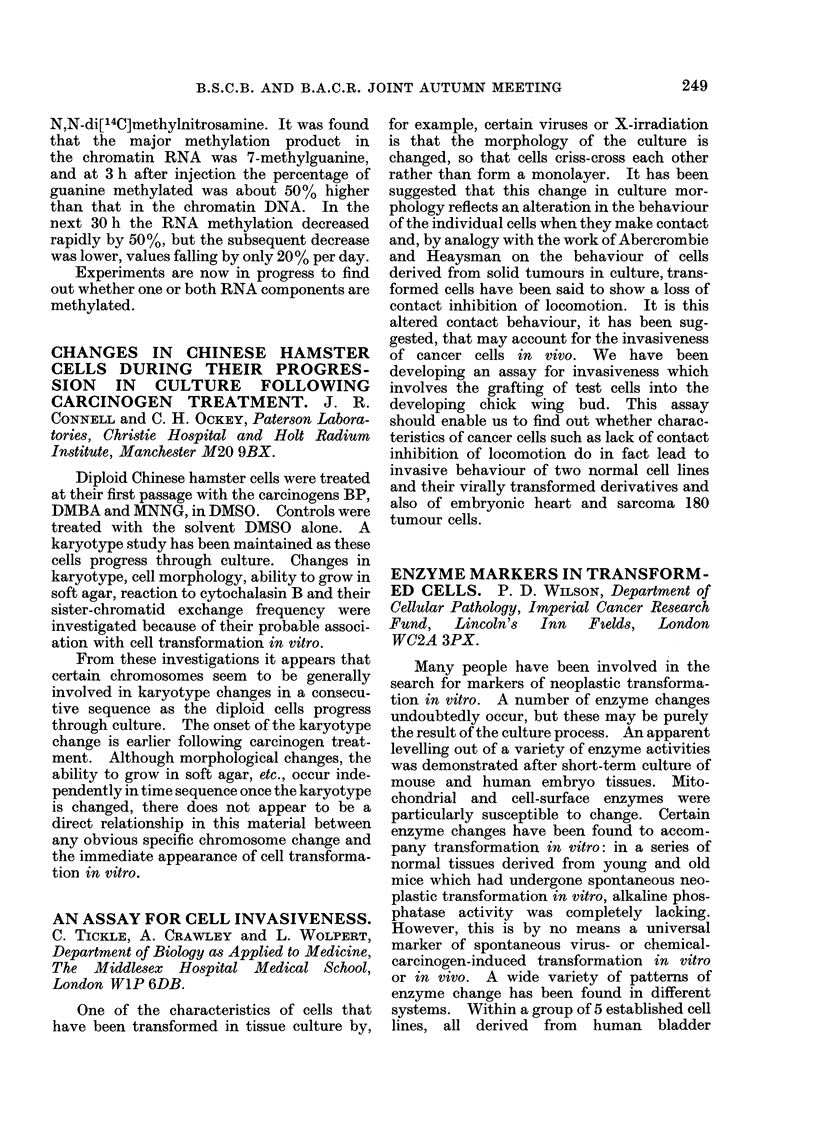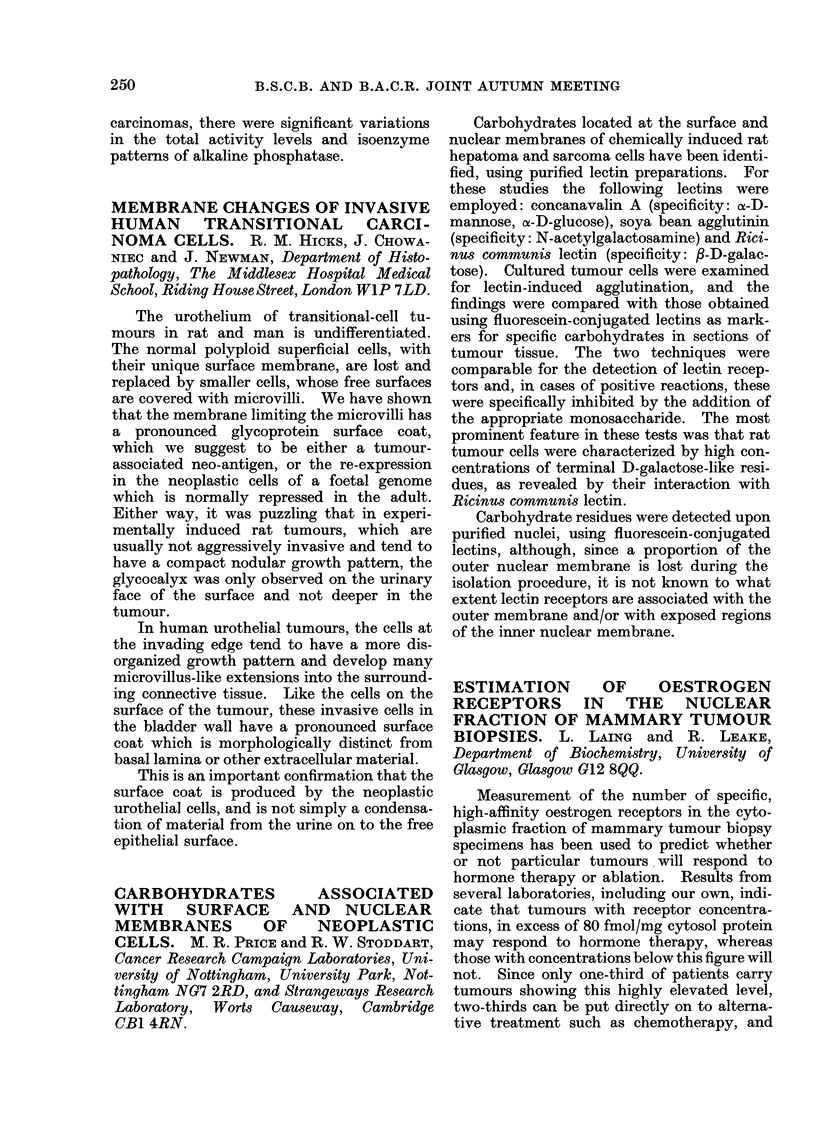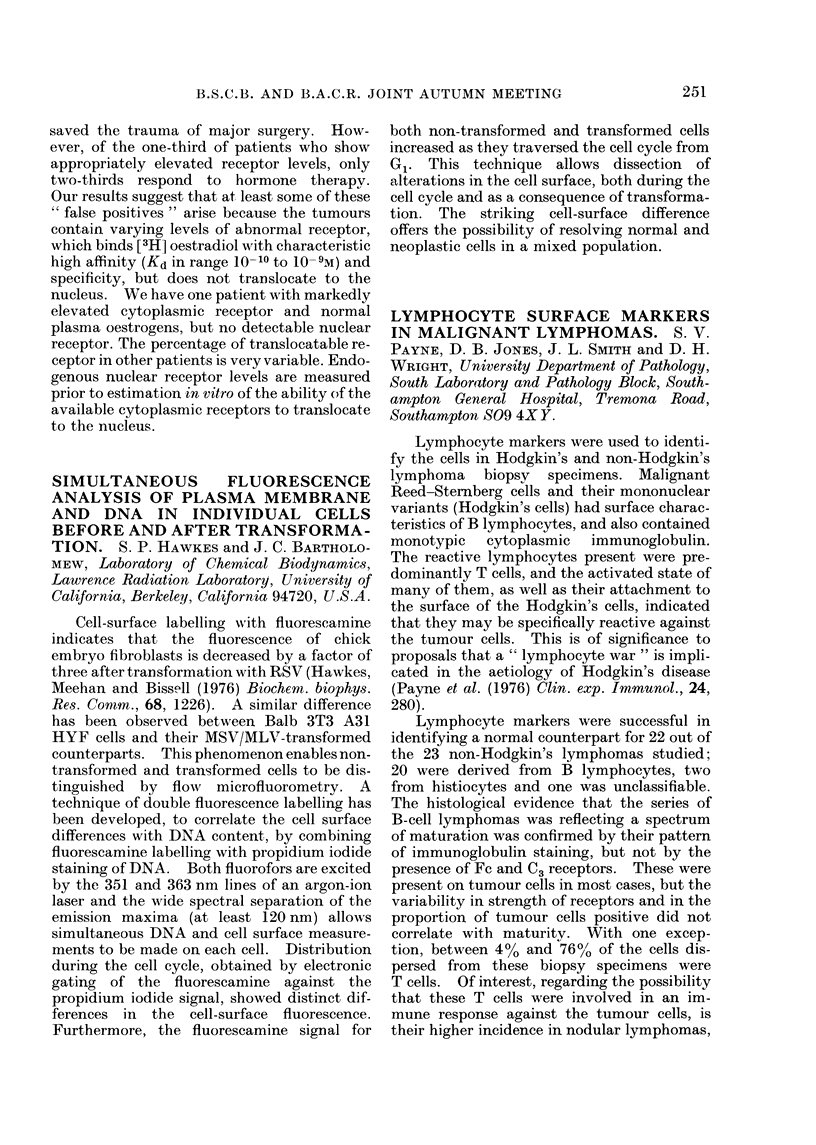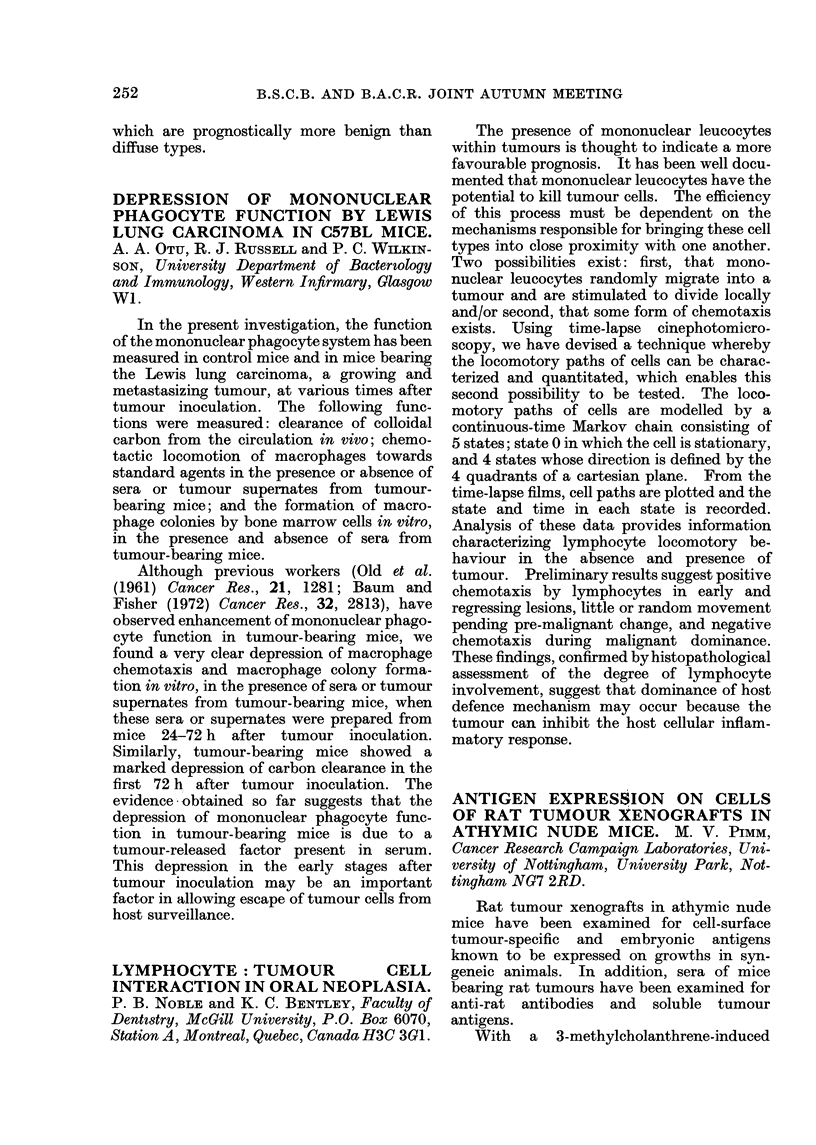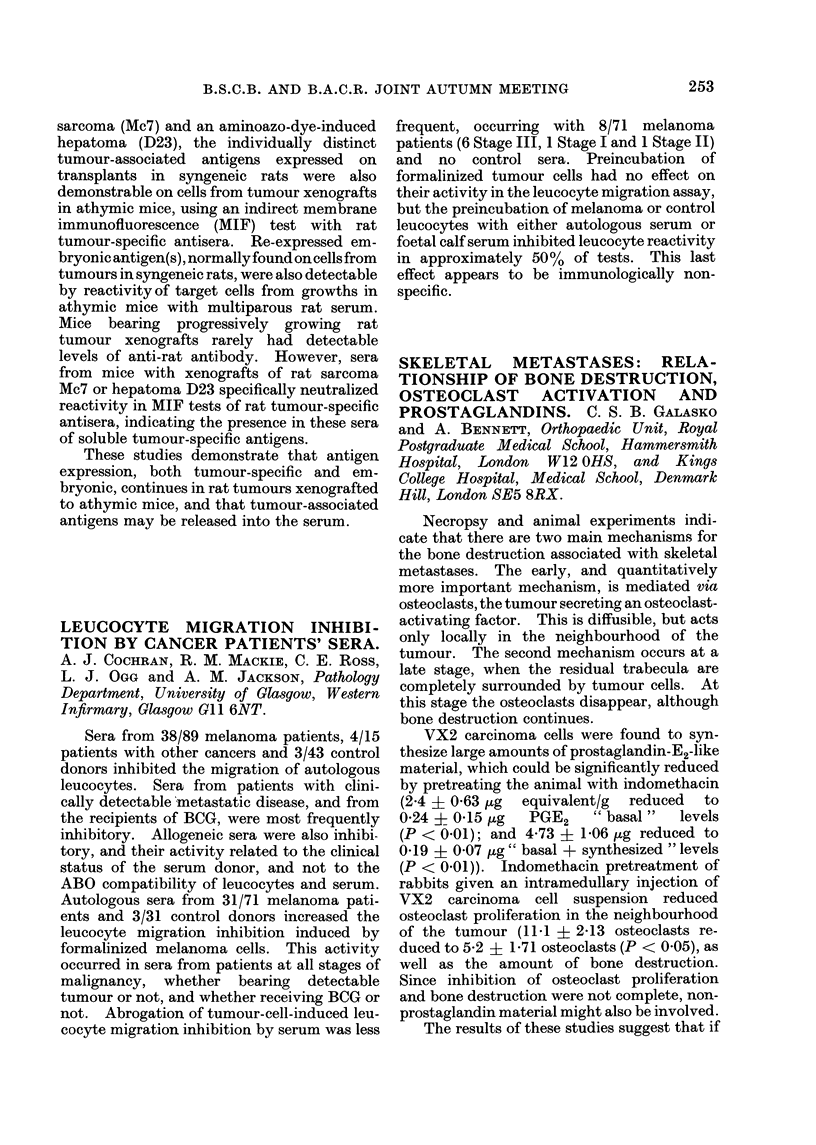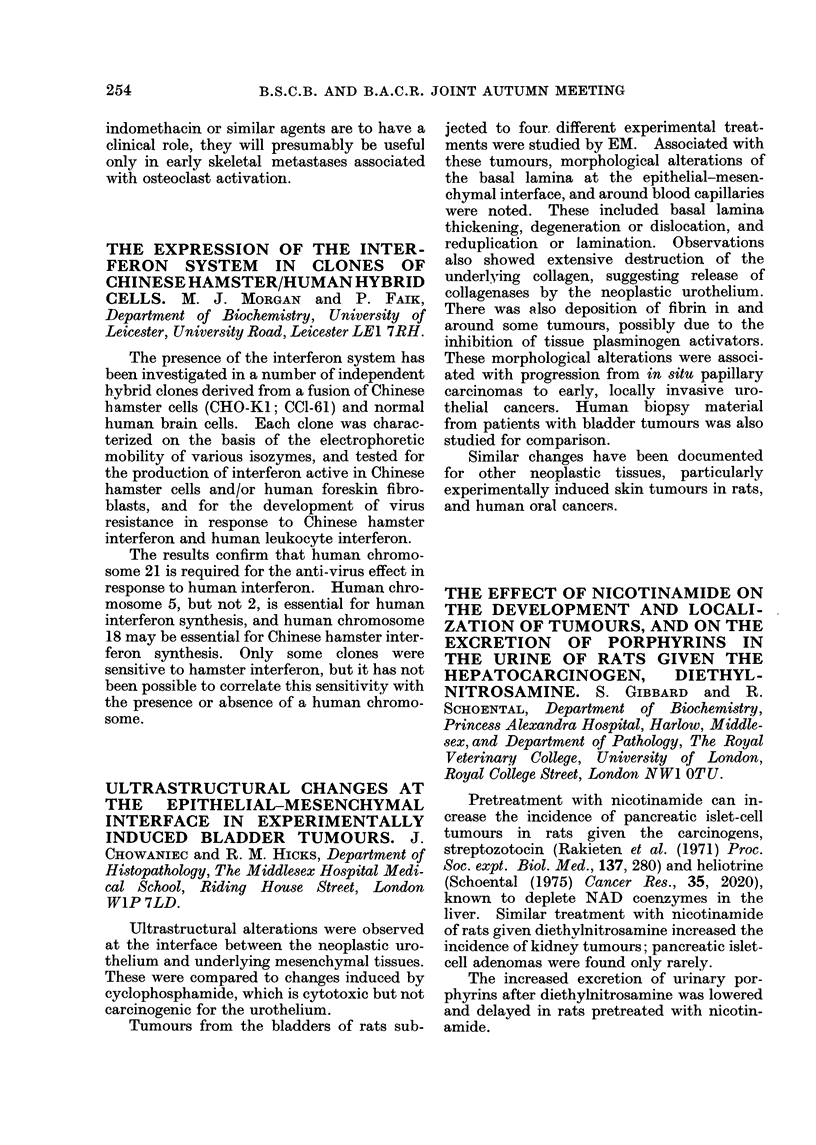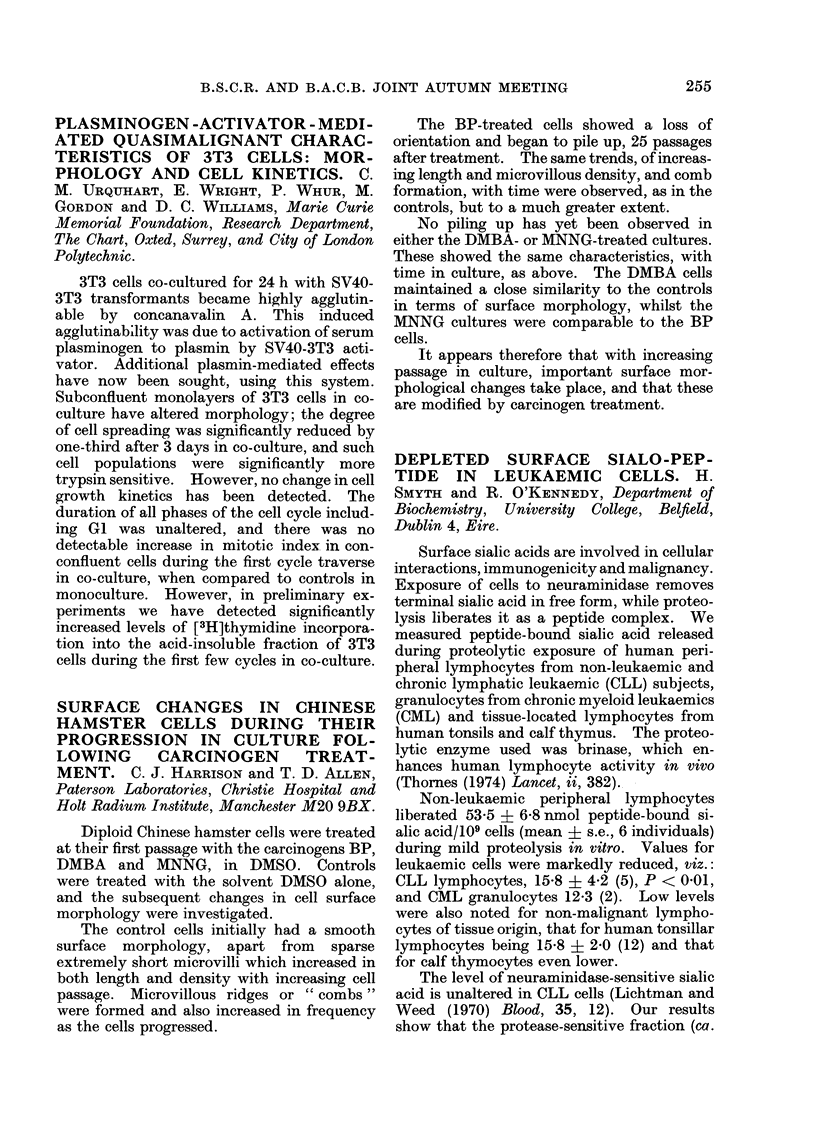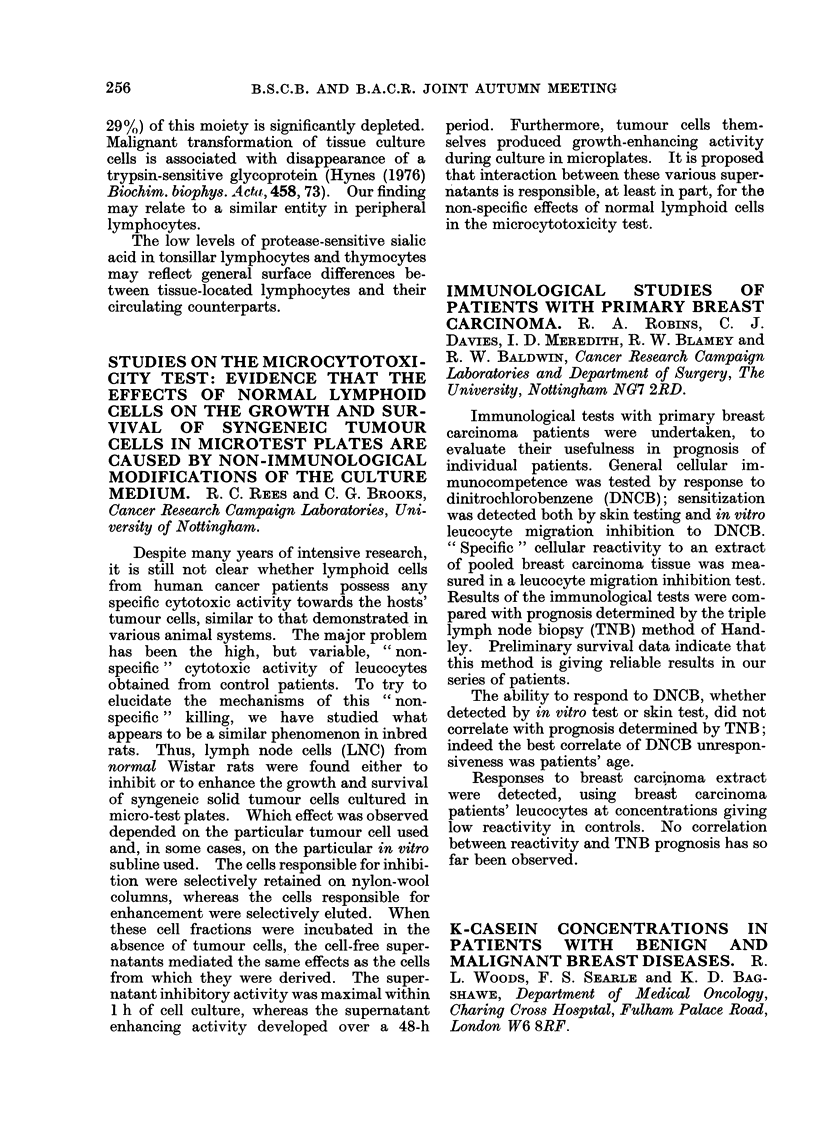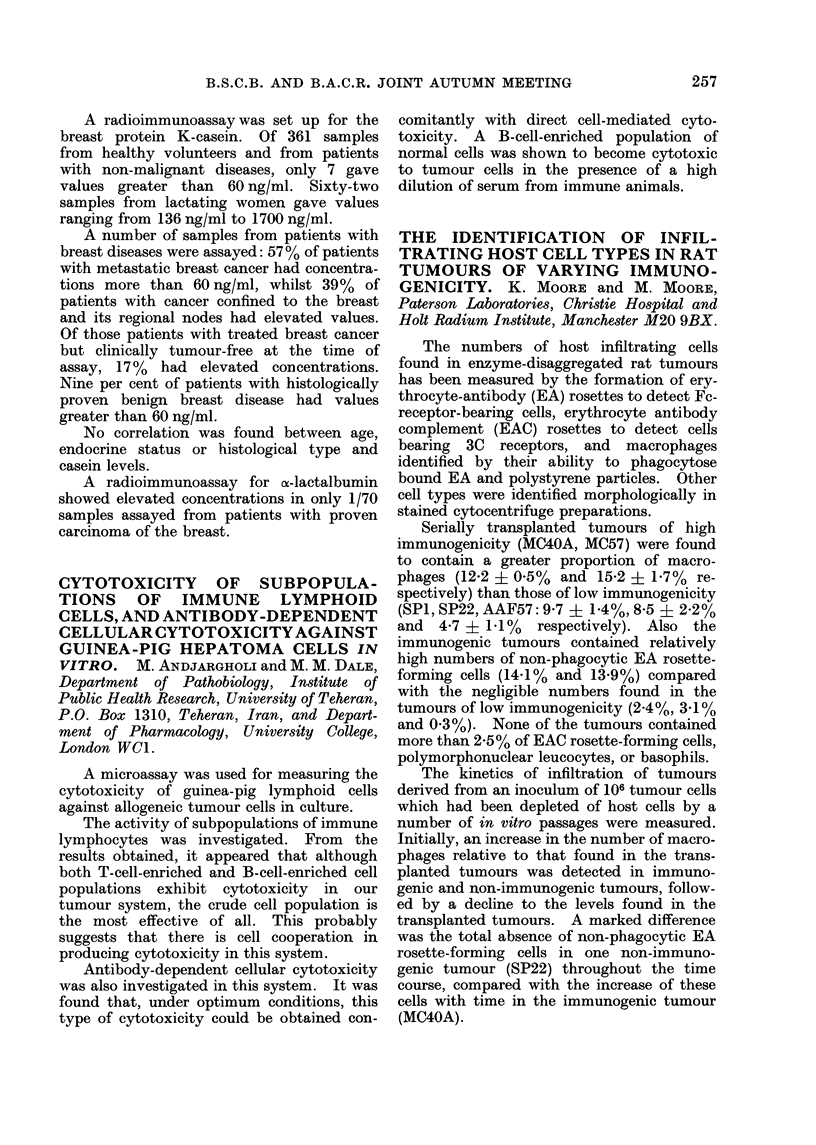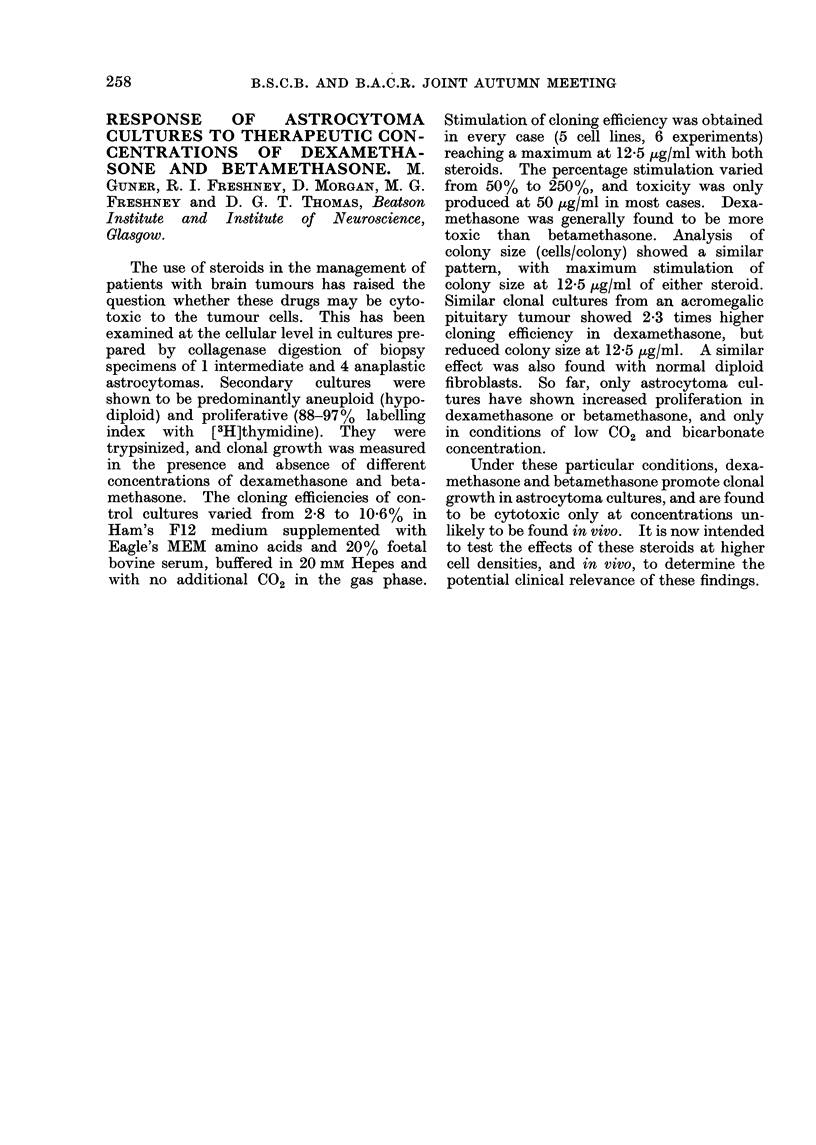# B.A.C.R. and B.S.C.B. Joint Autumn Meeting

**Published:** 1977-02

**Authors:** 


*Br. J. Cancer* (1977) **35**, 242

### BRITISH SOCIETY FOR CELL BIOLOGY
### AND
### BRITISH ASSOCIATION FOR CANCER RESEARCH

**Joint Autumn Meeting—Edward Lewis Theatre**
**Middlesex Hospital Medical School, London**

**28–30 September 1976**

---

### CELLULAR ASPECTS OF NEOPLASIA

---

### Titles of Symposia and Abstracts of Members' Proffered Papers

### SYMPOSIA

### I GENETIC NATURE OF NEOPLASIA

R. BASERGA, *Temple University School of Medicine, Philadelphia, Pennsylvania* 19140, *U.S.A.* (*Searle Lecturer*).

NUCLEAR CONTROL OF CELL PROLIFERATION.

J. CAIRNS, *Imperial Cancer Research Fund, Burtonhole Lane, London NW*7.

A PROMOTIONAL MODEL FOR THE PRODUCTION OF LUNG CANCER BY SMOKING.

J. F. WATKINS, *Department of Medical Microbiology, Welsh National School of Medicine, University of Wales, Heath Park, Cardiff CF*4 4*XN*.

THE ROLE OF CELL LINES DERIVED FROM HUMAN TUMOURS IN THE STUDY OF NEOPLASIA.

### II NEOPLASIA AND CYTODIFFERENTIATION

L. G. LAJTHA, *Paterson Laboratories, Christie Hospital and Holt Radium Institute, Manchester M*20 9*BX*.

PROLIFERATION CONTROL IN HAEMOPOIESIS.

P. R. HARRISON, *Wolfson Laboratory for Molecular Pathology, The Beatson Institute for Cancer Research, Garscube Estate, Bearsden Road, Glasgow C*61.

ANALYSIS OF ERYTHROPOIESIS AT THE MOLECULAR LEVEL.

M. J. EVANS, *Department of Anatomy and Embryology, University College London, London WC*1*E* 6*BT*.

CYTODIFFERENTIATION OF TERATOCARCINOMA CELLS.

### III NEOPLASTIC TRANSFORMATION

R. DULBECCO, *Imperial Cancer Research Fund, P.O. Box* 123, *Lincoln's Inn Fields, London WC*2*A* 3*PX*.

VIRAL ROLE IN CELL TRANSFORMATION BY THE SMALL DNA-CONTAINING VIRUSES, POLYOMA AND SV40.

R. WEISS, *Imperial Cancer Research Fund, P.O. Box* 123, *Lincoln's Inn Fields, London WC*2*A* 3*PX*.

THE ONCOGENE CONCEPT.

H. ROZENGURT, M. GRIMALDI and H. BOURNE, *Imperial Cancer Research Fund, P.O. Box* 123, *Lincoln's Inn Fields, London WC*2*A* 3*PX*.

AN 18,000 K-POLYPEPTIDE INDUCES EARLY EVENTS AND STIMULATES DNA SYNTHESIS IN CULTURED CELLS.

### IV MEMBRANE DIFFERENTIATION, CELL SURFACE ANTIGENS AND RECEPTOR SITES IN NEOPLASIA

R. W. STODDART, *Strangeuays Research Laboratory, Wort's Causeway, Cambridge CB*1 4*RN*.

MODIFICATIONS OF MEMBRANE GLYCOPROTEIN IN NEOPLASTIC DISEASE.

M. J. EMBLETON, *Cancer Research Campaign Laboratories, University of Nottingham, University Park, Nottingham NG*7 2*RD*.

CELL-SURFACE ANTIGENS ASSOCIATED WITH CHEMICALLY TRANSFORMED CELLS.

### V CELLULAR AND HUMORAL RESPONSES IN NEOPLASIA

G. KLEIN, *Department of Tumor Biology, Karolinska Institute, S* 104 01 *Stockholm* 60, *Sweden*.

CELLULAR AND HUMORAL IMMUNE REACTIONS IN RELATION TO THE THREE EPSTEIN–BARR VIRUS (EBV) ASSOCIATED HUMAN DISEASES (MONONUCLEOSIS, BURKITT'S LYMPHOMA AND NASOPHARYNGEAL CARCINOMA).

N. A. MITCHISON, *Imperial Cancer Research Fund, Tumour Immunology Unit, P.O. Box* 123, *Lincoln's Inn Fields, London WC*2*A* 3*PX*.

REGULATION OF THE IMMUNE RESPONSE TO CELL SURFACE ANTIGENS.

A. C. ALLISON, J. FERLUGA and H. U. SCHORLEMMER, *Clinical Research Centre, Watford Road, Harrow, Middlesex HA*1 3*UJ*.

LYMPHOCYTES AND MACROPHAGES IN ANTI-TUMOUR IMMUNITY.

### ABSTRACTS OF MEMBERS' PROFFERED PAPERS

### LETHAL RESPONSE TO DNA-DAMAGING AGENTS IN A VARIETY OF HUMAN FIBROBLAST CELL STRAINS.

C. F. Arlett, *MRC Cell Mutation Unit, University of Sussex, Falmer, Brighton, BN1 9QG.*

Human syndromes such as xeroderma pigmentosum (XP) and ataxia telangiectasia (AT) exhibit mutagen sensitivity at the level of the whole animal and at the cellular level. In the case of XP, at least 5 complementation groups have been observed for the known defect in excision repair of DNA lesions induced by UV light. The excision-proficient XPs (variants) have a defect in post-replication repair. At least 3 classes of AT cells, all sensitive to $\gamma$ irradiation, are known. There are those without and those with an endonuclease defect. Amongst the latter, two complementation groups have been observed.

The cross-sensitivity pattern for cell survival for cell strains from AT, XP, a sun-sensitive child (11961), Fanconi's anaemia and progeria is shown in the Table.

Table

| Cell strain | UV 254 nm | UV 310 nm | NHAAF | EMS | GAMMA | MITO-C | MMS |
|---|---|---|---|---|---|---|---|
| Xeroderma pigmentosum | +++ | ++ | ++ | − | − | − | |
| XP variant | + | − | | | − | | − |
| 11961 | ++ | ++ | − | ++ | − | − | − |
| Ataxia telangiectasia | − | − | − | | +++ | − | − |
| Fanconi's anaemia | − | | | | + | +++ | |
| Progeria | | | | | + | | |

The results indicate that:

(1) There is no overlap for cell killing between XP and AT, and that therefore their endonucleases are distinctive.

(2) 11961 with no known repair defect is *NOT* xeroderma pigmentosum (possibly Cockayne's syndrome).

(3) The limited overlap suggests: (a) different repair modes for each type of lesion; therefore normal individuals have a variety of repair capacities; (b) cell strains available to detect specific classes of DNA-damaging agents.

### RADIOSENSITIVITY IN ATAXIA TELANGIECTASIA.

A. M. R. Taylor, J. A. Metcalfe, J. M. Oxford and D. G. Harnden, *Department of Cancer Studies, The Medical School, University of Birmingham, Birmingham B15 2TJ.*

Patients with the recessively inherited syndrome ataxia telangiectasia (AT) show a high incidence of malignant disease, particularly of the reticuloendothelial system. This susceptibility may be related to the occurrence of spontaneous chromosome aberrations also seen in these patients. A recently confirmed clinical finding has been the unusual sensitivity to ionizing radiation of these patients (Cunliffe *et al.* (1975) *Br. J. Radiol.*, **48**, 374). Cell survival studies on AT skin fibroblasts following exposure to $\gamma$-rays and the effects of X-rays on lymphocyte chromosomes have shown that the radiosensitivity can be observed at the cellular level (Taylor *et al.* (1975) *Nature*, **258**, 427, and (1976) *Nature*, **260**, 441). Results from the cytogenetic work, together with *in vitro* enzymatic assay work (Paterson *et al.* (1976) *Nature*, **260**, 444) are consistent with the hypothesis that there is in AT a defect in the repair of bases damaged by ionizing radiation.

### DNA REPAIR AND MUTAGENESIS IN MAMMALIAN CELLS.

M. Fox, *Paterson Laboratories, Christie Hospital and Holt Radium Institute, Manchester M20 9BX.*

Considerable evidence now suggests that mutations to resistance to 8-azaguanine or 6-thioguanine are in the structural gene for HGPRT, when selection is under welldefined conditions. Linear dose-response relationships have been demonstrated for induced mutation in V79 cells. Both strong and weak carcinogens are mutagenic, but mutagenic potency expressed in terms of mutants induced/lethal hit cannot be correlated directly with carcinogenicity. Studies on cell lines of different sensitivity to cell killing, and use of the repair inhibitor caffeine, have provided evidence for a caffeine-insensitive "error prone" repair process in human and rodent cells. This process appears to operate on a wide range of DNA lesions (possibly those causing a similar amount of helical distortion). Thus we conclude that mutagenesis in mammalian cells is a consequence of "repair errors" and is largely independent of the detailed chemical nature of the DNA lesion.

**EFFECT OF DIMETHYLNITROSAMINE ON DNA REPLICATION AND DNA POLYMERASE IN LIVER REGENERATING AFTER PARTIAL HEPATECTOMY.** V. M. Craddock, *MRC Toxicology Unit, Medical Research Council Laboratories, Woodmansterne Road, Carshalton, Surrey SM5 4EF.*

A single treatment with DMN induces hepatocellular carcinoma, if given to adult rats during the period of restorative hyperplasia following partial hepatectomy, but not when given to intact animals. Tumour incidence is higher when DMN is given during the period of maximum DNA synthesis (at 24 h) than when given in the pre-replicative stage (at 6 h). Hence it appeared possible that replication of DNA damaged by the carcinogen could be a relevant event in initiation of cancer.

DNA replication *in vivo* was measured after administration of DMN at 6 h or at 24 h. Although DMN caused a dose-dependent inhibition of synthesis, more replication occurred during the period when methylated bases formed by reaction with the carcinogen were known to be present in DNA, when treatment was at 24 h than at 6 h. DMN given at 6 h resulted in a dose-dependent inhibition of induction of DNA polymerase, assayed *in vitro*. Parallel experiments were carried out with MMS, a non-hepatocarcinogenic methylating agent. The results suggest that when methylating agents are given early in the pre-replicative stage, damage to DNA inhibits induction of polymerase, and therefore extensive replication of DNA cannot occur until the damage has been repaired. When the alkylating agents are given at 24 h, by which time DNA polymerase has already been induced, the enzyme is not inhibited, and replication of changed DNA is likely to take place.

**SPONTANEOUS AND INDUCED PATTERNS OF THE EPSTEIN-BARR VIRUS (EBV) CYCLE IN HUMAN LYMPHOMA CELL LINES AND THEIR SOMATIC CELL HYBRIDS.** G. B. Clements and G. Klein, *Department of Pathology, University of Glasgow, Western Infirmary, Glasgow G11 6NT and Department of Tumor Biology, Karolinska Institute, S-10401 Stockholm 60, Sweden.*

Regulation of the expression of early antigen (EA) and virus capsid antigen (VCA) both spontaneously and after treatment with iododeoxyuridine (IUDR) or $P_3$HR-1-virus were studied in two somatic cell hybrids between human lymphoma lines. The hybrid 8A was derived from crossing the non-producer Raji with the spontaneous producer Daudi. The second hybrid, 83, was produced by fusing Raji with the EBV-genome-negative B-Lymphoma line BJAB.

The studies indicate that:

1. Spontaneous production of EA and VCA is regulated by controls differing from those of $P_3$HR-1 virus or IUDR-induced EA synthesis.
2. EA and VCA have independent restriction mechanisms, although EA production is required for VCA synthesis.
3. Inducibility of EA synthesis by $P_3$HR-1 virus and by IUDR appear to be under the influence of at least partially identical controls.

This system provides evidence about the nature of the precise regulatory mechanisms characteristic of individual cell lines that control expression of EBV-specified genetic information in human lymphoma cells. EBV DNA (usually multiple copies per cell) is present in virtually all established human lymphoblastoid and lymphoma cell lines and also in the tumour cells of nasopharyngeal carcinoma and most cases of Burkitt's lymphoma. The use of somatic cell hybrids provides an avenue by which the relationship between the presence in a cell of EBV-specified genetic information and malignancy may be investigated.

**THE EFFECT OF ALKYLATING AGENTS ON NUCLEAR POLY (ADP-RIBOSE) METABOLISM.** M. I. DAVIES, H. HALLDORSSON, S. SHALL and G. J. SKIDMORE, *Biochemistry Laboratory, School of Biological Sciences, University of Sussex, Brighton BN1 9QG.*

Several alkylating agents, particularly the substituted nitrosoureas, are used as anti-tumour drugs. These compounds have, in addition, mutagenic and carcinogenic activity. Decreases in the levels of the coenzyme NAD have been noted in animals treated with these drugs. Whether this is due to decreased biosynthesis or to increased degradation is unclear.

We have examined the response of NAD metabolism in mouse leukaemia cells to a toxic dose of streptozotocin—the glucose derivative of N-methyl-N-nitrosourea. The NAD level drops rapidly to 50% of control in 4 h, rising again to regain control levels after 8–12 h. The transience of the effect seems chiefly due to the instability of the drug in aqueous solution. The effect is not due to metabolites, for example cyanates, generated in the medium.

The cellular levels of NAD in L1210 leukaemia cells can be lowered to 10% of normal without loss of viability, by growing the cells in nicotinamide-free medium. The drop in NAD level is not the cause of the cytotoxicity.

Nicotinamide administered with streptozotocin will prevent the drop in cellular NAD level, but so will 5-methylnicotinamide and theophylline, which are not precursors of NAD. These latter are competitive inhibitors of poly(ADP-ribose) polymerase, the major enzyme of NAD degradation. Addition of the inhibitors actually increases the cytotoxicity of streptozotocin.

We suggest that some of the cytotoxic effects of alkylating agents are mediated by effects on chromatin-bound poly(ADP-ribose).

**CYTODIFFERENTIATION IN HUMAN ACUTE MYELOBLASTIC LEUKAEMIA.** S. LAN, E. A. MCCULLOCH and J. E. TILL, *Ontario Cancer Institute, Toronto, Canada.*

A procedure for establishing lineage relationships in haemopoietic differentiation is to measure correlation coefficients between different cell classes in clonal populations. Acute myeloblastic leukaemia (AML) can be regarded as a " clonal haemopathy ", arising from a neoplastic pluripotent stem cell. Thus the haemopoietic population in each patient can serve as a clone in which to measure such correlations. For this purpose, in addition to morphologically recognizable cells, a number of early precursors have been identified by developmental assays using cell cultures. For erythropoiesis, these include erythropoietin-dependent burst-forming units (BFU-E) recognized by the formation of large erythroid growth units after 14 days in culture, and erythropoietin-dependent colony-forming units (CFU-E) assayed by colony counts on Day 7. For granulopoiesis, progenitors (CFU-C) are assayed by granulopoietic colony formation dependent on appropriate stimulating molecules. In 22 marrow specimens from 16 patients with AML, significant correlations were observed as follows: BFU-E *vs* CFU-E ($P < 0{\cdot}001$); BFU-E *vs* platelets ($P < 0{\cdot}01$); BFU-E *vs* CFU-C ($P < 0{\cdot}01$). No significant correlation was observed between the BFU-E, CFU-E or CFU-C and erythroblasts or granulocytes. Such data suggest that tests for correlations may be used to investigate cell lineage relationships in AML, and predict that numbers of pluripotent leukaemic stem cells should correlate strongly with numbers of closely-related cell classes such as BFU-E and CFU-C. This approach should be valuable in the development and validation of assays for putative pluripotent leukaemic stem cells.

**STUDIES ON *IN VITRO* DIFFERENTIATION OF ACUTE MYELOGENOUS LEUKAEMIA CELLS.** F. R. BALKWILL and R. T. D. OLIVER, *I.C.R.F. Department of Medical Onocology, St Bartholomew's Hospital, London EC1A 7BE.*

Peripheral blood and bone marrow cells from patients with acute myelogenous leukaemia (AML) show increases in [$^3$H] thymidine ($^3$HThy) incorporation and absolute cell number, when grown in short-term liquid culture without the addition of an exogenous source of stimulating factor. Cultures from 68 out of 78 (87%) AML patients showed $^3$HThy incorporation greater than 1000 ct/min/$2 \times 10^5$ cells after 96 h (range 1500–46,000 ct/min, mean 10,600). A similar range was also seen in bone marrow cultures from 31 patients (mean 11,330) and there was a good correlation between peripheral blood and bone marrow results. In contrast, studies on peripheral blood cells from patients with acute lymphoblastic leukaemia (ALL) showed significantly less incorporation (mean 1600 ct/min: $n = 16$) though this was higher than that of normal peripheral blood (mean 110 ct/min: $n = 4$). Detailed morphological studies demonstrated wide variation from patient to patient. 80% of cultures developed plastic-adherent trypsin-resistant fusiform cells, with functional surface membrane and cytochemical characteristics of macrophages. Time-course studies demonstrated that these cells were derived by differentiation *in vitro* from more primitive precursor cells. Quantitative assessment of the capacity for AML cells to differentiate into macrophages in 83 patients was shown to be a strong indicator of response to treatment.

Detailed investigation, using specific monocyte and granulocyte cytochemical stains on 15-day cultures from 24 patients, showed evidence of monocyte differentiation in 16/24 patients, though 7 of these showed evidence of both monocyte and granulocyte differentiation, suggesting either duoclonality or initiation of the disease in a common stem cell. A further 4 cultures showed evidence of granulocyte differentiation alone, and 4 no evidence of either monocyte or granulocyte differentiation. This extreme heterogeneity makes it extremely unlikely that a unitary hypothesis could explain the pathogenesis of this disease.

**APPEARANCE OF ABNORMAL CHROMOSOMES ASSOCIATED WITH SPONTANEOUS AND CHEMICALLY INDUCED TRANSFORMATION OF CULTURED CHINESE HAMSTER CELLS.** D. J. Kirkland and S. Venitt, *Institute of Cancer Research, The Royal Marsden Hospital, Fulham Road, London SW3 6JJ.*

The chromosomes of cloned Chinese hamster prostate and kidney cells, and of 10 subclones derived from them, have been examined for structural and numerical changes. By standard tissue culture criteria the 2 parental clones and the 2 subclones were normal, whereas 8 subclones were transformed, either spontaneously or by chemical induction.

A telocentric chromosome additional to the diploid complement was found in only 1% of the parental kidney clone, but was found in over 98% of the cells of 6 transformed kidney subclones. One subclone was isolated from an untreated culture, the others from 3-methyl-cholanthrene-treated cultures. Giemsa banding demonstrated this chromosome to be the centromere and long (q) arms of the Number 4 chromosome in every case.

A consistent telocentric chromosome, absent from the parental prostate clone and 2 normal subclones, was found in over 97% of the cells of 2 transformed subclones isolated from N-methyl-N-nitrosourea-treated cultures. This chromosome was found to be the centromere and most of the short (p) arms of the Number 4 chromosome in every case.

The discovery of specific chromosomal changes associated with spontaneously or chemically transformed cells has seldom been reported. The specific abnormalities reported here might not have been detected had the cells not been grown in a stabilizing medium suggested by Yerganian and Lavappa (in A. Hollaender (Ed.) (1971) *Chemical Mutagens, Principles and Methods for their Detection*, Vol. 2, p. 387, for the maintenance of Chinese hamster cell karyotypes.

**ON THE CRITICAL DIFFERENCE BETWEEN NORMAL AND CANCER CELLS, AND THE POSSIBLE ORIGIN OF THE HAYFLICK LIMIT.** R. J. Goldacre, *Chester Beatty Research Institute, Fulham Road, London SW3 6JB.*

While it has been widely believed in the past that cancer is due to mutation, there is now reason to think that cancer may result from a process akin to differentiation, *i.e. maldifferentiation*. Is the cancer genome different from the normal? There are no known general biochemical differences between normal and cancer cells. Properties distinguishing cancer cells from corresponding normal cells (including biochemical and cell surface changes) can always be matched singly in some normal cell or other—as if all these " cancer " properties are *needed* by some normal cell during normal life.

It seems as if some intracellular control mechanisms have been delegated in normal cells to act *between* cells, while the cancer cell has retained them within itself: a process requiring rearrangement of molecules but not generation of new ones. This delegation appears to have a bearing on the Hayflick limit.

Cancer cells resemble protozoa which can live independent lives. When metazoa evolved, genetic instructions had to be provided to the cell to adhere, pass growth-controlling information from cell to cell, *etc.* It is likely that, in mammals, repressed genes still persist for unicellular life (as persists the genetic code from that time—the same code for plants, animals and bacteria). Thus the opportunity to (mal)differentiate back may persist. Conversely Papaionnou *et al.* ((1975) *Nature*, **258**, 70) have recently reported the differentiation of genetically marked teratocarcinoma cells, fused with a blastula, into normal tissue.

Nuclei were exchanged between 5 different pairs of normal and cancer cells in the mouse; wherever it has been possible to make an assessment of malignancy in the resulting cells, by tumour formation or cell surface properties, the malignancy corresponded to that of the cytoplasm and not of the nucleus.

### SELECTIVE INHIBITION OF THE OUTGROWTH OF EPSTEIN–BARR VIRUS-CARRYING CELL LINES FROM LEUCOCYTES OF INFECTIOUS MONONUCLEOSIS PATIENTS.

A. B. Rickinson and M. A. Epstein, *Department of Pathology, The Medical School, University of Bristol, Bristol BS8 1TD*

The leucocytes of infectious mononucleosis (IM) patients readily gave rise to Epstein–Barr (EB) virus-genome-containing lymphoid cell lines *in vitro*. The view that the virus-carrying cells in IM blood are actually transformed by the virus *in vivo* has recently been challenged by the observation that transformed foci arise in IM leucocyte cultures, not primarily through a process of direct outgrowth, but by a quite different two-step mechanism; thus cells harbouring the virus *in vivo* enter a productive cycle *in vitro* and release virus particles, which then go on to infect lymphocytes co-resident in the culture, transforming them into lymphoblastoid cell lines. The present experiments seek to determine whether this two-step mechanism is wholly responsible for the establishment of cell lines from IM leucocytes, or whether it serves to mask the direct *in vitro* outgrowth of cells already transformed by EB virus *in vivo*.

The first experiments show that disodium phosphonoacetate (PA), when present in culture medium at a concentration of 50 μg/ml, strongly inhibits the replication of EB virus in cells of a producer line, but does not seriously affect either the growth rate of virally transformed cells, or the transformation of foetal lymphocytes following their infection by the virus *in vitro*. The second experiments show that when IM leucocytes are cultured in medium containing 50 μg PA/ml, that is under conditions which selectively block the two-step mechanism, the establishment of EB virus-genome-containing cell lines is abolished. These results confirm the exclusive role proposed for the two-step mechanism in the development of such cell lines, and argue against the existence in IM blood of cells already transformed by EB virus and capable of independent *in vitro* growth.

### STUDIES ON LIVER CHROMATIN RNA FROM RATS TREATED WITH NN-DI[$^{14}$C]METHYLNITROSAMINE.

A. I. Galbraith and R. F. Itzhaki, *Paterson Laboratories, Christie Hospital and Holt Radium Institute, Manchester M20 9BX.*

As part of a study of the effect of carcinogens on chromatin (Saffhill *et al.* (1974) *Nature* **248**, 153; Cooper and Itzhaki (1975) *Biochem. biophys. Acta*, **407**, 263; Cooper *et al.* (1975) *Chem. Biol. Interactions*, **11**, 483) we have examined the methylation of liver chromatin RNA after treatment of rats with dimethylnitrosamine. We have previously shown that the RNA consists of a stable, low mol. wt component and a rapidly synthesized component of higher mol. wt, probably comprising nascent messenger RNA.

Chromatin was prepared from rat livers 3–72 h after injection of the hepatocarcinogen

N,N-di[$^{14}$C]methylnitrosamine. It was found that the major methylation product in the chromatin RNA was 7-methylguanine, and at 3 h after injection the percentage of guanine methylated was about 50% higher than that in the chromatin DNA. In the next 30 h the RNA methylation decreased rapidly by 50%, but the subsequent decrease was lower, values falling by only 20% per day.

Experiments are now in progress to find out whether one or both RNA components are methylated.

**CHANGES IN CHINESE HAMSTER CELLS DURING THEIR PROGRESSION IN CULTURE FOLLOWING CARCINOGEN TREATMENT.** J. R. CONNELL and C. H. OCKEY, *Paterson Laboratories, Christie Hospital and Holt Radium Institute, Manchester M20 9BX.*

Diploid Chinese hamster cells were treated at their first passage with the carcinogens BP, DMBA and MNNG, in DMSO. Controls were treated with the solvent DMSO alone. A karyotype study has been maintained as these cells progress through culture. Changes in karyotype, cell morphology, ability to grow in soft agar, reaction to cytochalasin B and their sister-chromatid exchange frequency were investigated because of their probable association with cell transformation *in vitro*.

From these investigations it appears that certain chromosomes seem to be generally involved in karyotype changes in a consecutive sequence as the diploid cells progress through culture. The onset of the karyotype change is earlier following carcinogen treatment. Although morphological changes, the ability to grow in soft agar, *etc.*, occur independently in time sequence once the karyotype is changed, there does not appear to be a direct relationship in this material between any obvious specific chromosome change and the immediate appearance of cell transformation *in vitro*.

**AN ASSAY FOR CELL INVASIVENESS.** C. TICKLE, A. CRAWLEY and L. WOLPERT, *Department of Biology as Applied to Medicine, The Middlesex Hospital Medical School, London W1P 6DB.*

One of the characteristics of cells that have been transformed in tissue culture by, for example, certain viruses or X-irradiation is that the morphology of the culture is changed, so that cells criss-cross each other rather than form a monolayer. It has been suggested that this change in culture morphology reflects an alteration in the behaviour of the individual cells when they make contact and, by analogy with the work of Abercrombie and Heaysman on the behaviour of cells derived from solid tumours in culture, transformed cells have been said to show a loss of contact inhibition of locomotion. It is this altered contact behaviour, it has been suggested, that may account for the invasiveness of cancer cells *in vivo*. We have been developing an assay for invasiveness which involves the grafting of test cells into the developing chick wing bud. This assay should enable us to find out whether characteristics of cancer cells such as lack of contact inhibition of locomotion do in fact lead to invasive behaviour of two normal cell lines and their virally transformed derivatives and also of embryonic heart and sarcoma 180 tumour cells.

**ENZYME MARKERS IN TRANSFORMED CELLS.** P. D. WILSON, *Department of Cellular Pathology, Imperial Cancer Research Fund, Lincoln's Inn Fields, London WC2A 3PX.*

Many people have been involved in the search for markers of neoplastic transformation *in vitro*. A number of enzyme changes undoubtedly occur, but these may be purely the result of the culture process. An apparent levelling out of a variety of enzyme activities was demonstrated after short-term culture of mouse and human embryo tissues. Mitochondrial and cell-surface enzymes were particularly susceptible to change. Certain enzyme changes have been found to accompany transformation *in vitro*: in a series of normal tissues derived from young and old mice which had undergone spontaneous neoplastic transformation *in vitro*, alkaline phosphatase activity was completely lacking. However, this is by no means a universal marker of spontaneous virus- or chemical-carcinogen-induced transformation *in vitro* or *in vivo*. A wide variety of patterns of enzyme change has been found in different systems. Within a group of 5 established cell lines, all derived from human bladder carcinomas, there were significant variations in the total activity levels and isoenzyme patterns of alkaline phosphatase.

**MEMBRANE CHANGES OF INVASIVE HUMAN TRANSITIONAL CARCINOMA CELLS.** R. M. HICKS, J. CHOWANIEC and J. NEWMAN, *Department of Histopathology, The Middlesex Hospital Medical School, Riding House Street, London W1P 7LD.*

The urothelium of transitional-cell tumours in rat and man is undifferentiated. The normal polyploid superficial cells, with their unique surface membrane, are lost and replaced by smaller cells, whose free surfaces are covered with microvilli. We have shown that the membrane limiting the microvilli has a pronounced glycoprotein surface coat, which we suggest to be either a tumour-associated neo-antigen, or the re-expression in the neoplastic cells of a foetal genome which is normally repressed in the adult. Either way, it was puzzling that in experimentally induced rat tumours, which are usually not aggressively invasive and tend to have a compact nodular growth pattern, the glycocalyx was only observed on the urinary face of the surface and not deeper in the tumour.

In human urothelial tumours, the cells at the invading edge tend to have a more disorganized growth pattern and develop many microvillus-like extensions into the surrounding connective tissue. Like the cells on the surface of the tumour, these invasive cells in the bladder wall have a pronounced surface coat which is morphologically distinct from basal lamina or other extracellular material.

This is an important confirmation that the surface coat is produced by the neoplastic urothelial cells, and is not simply a condensation of material from the urine on to the free epithelial surface.

**CARBOHYDRATES ASSOCIATED WITH SURFACE AND NUCLEAR MEMBRANES OF NEOPLASTIC CELLS.** M. R. PRICE and R. W. STODDART, *Cancer Research Campaign Laboratories, University of Nottingham, University Park, Nottingham NG7 2RD, and Strangeways Research Laboratory, Worts Causeway, Cambridge CB1 4RN.*

Carbohydrates located at the surface and nuclear membranes of chemically induced rat hepatoma and sarcoma cells have been identified, using purified lectin preparations. For these studies the following lectins were employed: concanavalin A (specificity: $\alpha$-D-mannose, $\alpha$-D-glucose), soya bean agglutinin (specificity: N-acetylgalactosamine) and *Ricinus communis* lectin (specificity: $\beta$-D-galactose). Cultured tumour cells were examined for lectin-induced agglutination, and the findings were compared with those obtained using fluorescein-conjugated lectins as markers for specific carbohydrates in sections of tumour tissue. The two techniques were comparable for the detection of lectin receptors and, in cases of positive reactions, these were specifically inhibited by the addition of the appropriate monosaccharide. The most prominent feature in these tests was that rat tumour cells were characterized by high concentrations of terminal D-galactose-like residues, as revealed by their interaction with *Ricinus communis* lectin.

Carbohydrate residues were detected upon purified nuclei, using fluorescein-conjugated lectins, although, since a proportion of the outer nuclear membrane is lost during the isolation procedure, it is not known to what extent lectin receptors are associated with the outer membrane and/or with exposed regions of the inner nuclear membrane.

**ESTIMATION OF OESTROGEN RECEPTORS IN THE NUCLEAR FRACTION OF MAMMARY TUMOUR BIOPSIES.** L. LAING and R. LEAKE, *Department of Biochemistry, University of Glasgow, Glasgow G12 8QQ.*

Measurement of the number of specific, high-affinity oestrogen receptors in the cytoplasmic fraction of mammary tumour biopsy specimens has been used to predict whether or not particular tumours will respond to hormone therapy or ablation. Results from several laboratories, including our own, indicate that tumours with receptor concentrations, in excess of 80 fmol/mg cytosol protein may respond to hormone therapy, whereas those with concentrations below this figure will not. Since only one-third of patients carry tumours showing this highly elevated level, two-thirds can be put directly on to alternative treatment such as chemotherapy, and saved the trauma of major surgery. However, of the one-third of patients who show appropriately elevated receptor levels, only two-thirds respond to hormone therapy. Our results suggest that at least some of these " false positives " arise because the tumours contain varying levels of abnormal receptor, which binds [$^3$H] oestradiol with characteristic high affinity ($K_d$ in range $10^{-10}$ to $10^{-9}$M) and specificity, but does not translocate to the nucleus. We have one patient with markedly elevated cytoplasmic receptor and normal plasma oestrogens, but no detectable nuclear receptor. The percentage of translocatable receptor in other patients is very variable. Endogenous nuclear receptor levels are measured prior to estimation *in vitro* of the ability of the available cytoplasmic receptors to translocate to the nucleus.

### SIMULTANEOUS FLUORESCENCE ANALYSIS OF PLASMA MEMBRANE AND DNA IN INDIVIDUAL CELLS BEFORE AND AFTER TRANSFORMATION.

S. P. HAWKES and J. C. BARTHOLOMEW, *Laboratory of Chemical Biodynamics, Lawrence Radiation Laboratory, University of California, Berkeley, California* 94720, *U.S.A.*

Cell-surface labelling with fluorescamine indicates that the fluorescence of chick embryo fibroblasts is decreased by a factor of three after transformation with RSV (Hawkes, Meehan and Bissell (1976) *Biochem. biophys. Res. Comm.*, **68**, 1226). A similar difference has been observed between Balb 3T3 A31 HYF cells and their MSV/MLV-transformed counterparts. This phenomenon enables non-transformed and transformed cells to be distinguished by flow microfluorometry. A technique of double fluorescence labelling has been developed, to correlate the cell surface differences with DNA content, by combining fluorescamine labelling with propidium iodide staining of DNA. Both fluorofors are excited by the 351 and 363 nm lines of an argon-ion laser and the wide spectral separation of the emission maxima (at least 120 nm) allows simultaneous DNA and cell surface measurements to be made on each cell. Distribution during the cell cycle, obtained by electronic gating of the fluorescamine against the propidium iodide signal, showed distinct differences in the cell-surface fluorescence. Furthermore, the fluorescamine signal for both non-transformed and transformed cells increased as they traversed the cell cycle from $G_1$. This technique allows dissection of alterations in the cell surface, both during the cell cycle and as a consequence of transformation. The striking cell-surface difference offers the possibility of resolving normal and neoplastic cells in a mixed population.

### LYMPHOCYTE SURFACE MARKERS IN MALIGNANT LYMPHOMAS.

S. V. PAYNE, D. B. JONES, J. L. SMITH and D. H. WRIGHT, *University Department of Pathology, South Laboratory and Pathology Block, Southampton General Hospital, Tremona Road, Southampton SO9 4XY.*

Lymphocyte markers were used to identify the cells in Hodgkin's and non-Hodgkin's lymphoma biopsy specimens. Malignant Reed–Sternberg cells and their mononuclear variants (Hodgkin's cells) had surface characteristics of B lymphocytes, and also contained monotypic cytoplasmic immunoglobulin. The reactive lymphocytes present were predominantly T cells, and the activated state of many of them, as well as their attachment to the surface of the Hodgkin's cells, indicated that they may be specifically reactive against the tumour cells. This is of significance to proposals that a " lymphocyte war " is implicated in the aetiology of Hodgkin's disease (Payne *et al.* (1976) *Clin. exp. Immunol.*, **24**, 280).

Lymphocyte markers were successful in identifying a normal counterpart for 22 out of the 23 non-Hodgkin's lymphomas studied; 20 were derived from B lymphocytes, two from histiocytes and one was unclassifiable. The histological evidence that the series of B-cell lymphomas was reflecting a spectrum of maturation was confirmed by their pattern of immunoglobulin staining, but not by the presence of Fc and $C_3$ receptors. These were present on tumour cells in most cases, but the variability in strength of receptors and in the proportion of tumour cells positive did not correlate with maturity. With one exception, between 4% and 76% of the cells dispersed from these biopsy specimens were T cells. Of interest, regarding the possibility that these T cells were involved in an immune response against the tumour cells, is their higher incidence in nodular lymphomas, which are prognostically more benign than diffuse types.

### DEPRESSION OF MONONUCLEAR PHAGOCYTE FUNCTION BY LEWIS LUNG CARCINOMA IN C57BL MICE.

A. A. OTU, R. J. RUSSELL and P. C. WILKINSON, *University Department of Bacteriology and Immunology, Western Infirmary, Glasgow* W1.

In the present investigation, the function of the mononuclear phagocyte system has been measured in control mice and in mice bearing the Lewis lung carcinoma, a growing and metastasizing tumour, at various times after tumour inoculation. The following functions were measured: clearance of colloidal carbon from the circulation *in vivo*; chemotactic locomotion of macrophages towards standard agents in the presence or absence of sera or tumour supernates from tumour-bearing mice; and the formation of macrophage colonies by bone marrow cells *in vitro*, in the presence and absence of sera from tumour-bearing mice.

Although previous workers (Old *et al.* (1961) *Cancer Res.*, **21**, 1281; Baum and Fisher (1972) *Cancer Res.*, **32**, 2813), have observed enhancement of mononuclear phagocyte function in tumour-bearing mice, we found a very clear depression of macrophage chemotaxis and macrophage colony formation *in vitro*, in the presence of sera or tumour supernates from tumour-bearing mice, when these sera or supernates were prepared from mice 24–72 h after tumour inoculation. Similarly, tumour-bearing mice showed a marked depression of carbon clearance in the first 72 h after tumour inoculation. The evidence obtained so far suggests that the depression of mononuclear phagocyte function in tumour-bearing mice is due to a tumour-released factor present in serum. This depression in the early stages after tumour inoculation may be an important factor in allowing escape of tumour cells from host surveillance.

### LYMPHOCYTE : TUMOUR CELL INTERACTION IN ORAL NEOPLASIA.

P. B. NOBLE and K. C. BENTLEY, *Faculty of Dentistry, McGill University, P.O. Box* 6070, *Station A, Montreal, Quebec, Canada H3C 3G1*.

The presence of mononuclear leucocytes within tumours is thought to indicate a more favourable prognosis. It has been well documented that mononuclear leucocytes have the potential to kill tumour cells. The efficiency of this process must be dependent on the mechanisms responsible for bringing these cell types into close proximity with one another. Two possibilities exist: first, that mononuclear leucocytes randomly migrate into a tumour and are stimulated to divide locally and/or second, that some form of chemotaxis exists. Using time-lapse cinephotomicroscopy, we have devised a technique whereby the locomotory paths of cells can be characterized and quantitated, which enables this second possibility to be tested. The locomotory paths of cells are modelled by a continuous-time Markov chain consisting of 5 states; state 0 in which the cell is stationary, and 4 states whose direction is defined by the 4 quadrants of a cartesian plane. From the time-lapse films, cell paths are plotted and the state and time in each state is recorded. Analysis of these data provides information characterizing lymphocyte locomotory behaviour in the absence and presence of tumour. Preliminary results suggest positive chemotaxis by lymphocytes in early and regressing lesions, little or random movement pending pre-malignant change, and negative chemotaxis during malignant dominance. These findings, confirmed by histopathological assessment of the degree of lymphocyte involvement, suggest that dominance of host defence mechanism may occur because the tumour can inhibit the host cellular inflammatory response.

### ANTIGEN EXPRESSION ON CELLS OF RAT TUMOUR XENOGRAFTS IN ATHYMIC NUDE MICE.

M. V. PIMM, *Cancer Research Campaign Laboratories, University of Nottingham, University Park, Nottingham NG7 2RD.*

Rat tumour xenografts in athymic nude mice have been examined for cell-surface tumour-specific and embryonic antigens known to be expressed on growths in syngeneic animals. In addition, sera of mice bearing rat tumours have been examined for anti-rat antibodies and soluble tumour antigens.

With a 3-methylcholanthrene-induced sarcoma (Mc7) and an aminoazo-dye-induced hepatoma (D23), the individually distinct tumour-associated antigens expressed on transplants in syngeneic rats were also demonstrable on cells from tumour xenografts in athymic mice, using an indirect membrane immunofluorescence (MIF) test with rat tumour-specific antisera. Re-expressed embryonic antigen(s), normally found on cells from tumours in syngeneic rats, were also detectable by reactivity of target cells from growths in athymic mice with multiparous rat serum. Mice bearing progressively growing rat tumour xenografts rarely had detectable levels of anti-rat antibody. However, sera from mice with xenografts of rat sarcoma Mc7 or hepatoma D23 specifically neutralized reactivity in MIF tests of rat tumour-specific antisera, indicating the presence in these sera of soluble tumour-specific antigens.

These studies demonstrate that antigen expression, both tumour-specific and embryonic, continues in rat tumours xenografted to athymic mice, and that tumour-associated antigens may be released into the serum.

### LEUCOCYTE MIGRATION INHIBITION BY CANCER PATIENTS' SERA.

A. J. Cochran, R. M. Mackie, C. E. Ross, L. J. Ogg and A. M. Jackson, *Pathology Department, University of Glasgow, Western Infirmary, Glasgow G11 6NT.*

Sera from 38/89 melanoma patients, 4/15 patients with other cancers and 3/43 control donors inhibited the migration of autologous leucocytes. Sera from patients with clinically detectable metastatic disease, and from the recipients of BCG, were most frequently inhibitory. Allogeneic sera were also inhibitory, and their activity related to the clinical status of the serum donor, and not to the ABO compatibility of leucocytes and serum. Autologous sera from 31/71 melanoma patients and 3/31 control donors increased the leucocyte migration inhibition induced by formalinized melanoma cells. This activity occurred in sera from patients at all stages of malignancy, whether bearing detectable tumour or not, and whether receiving BCG or not. Abrogation of tumour-cell-induced leucocyte migration inhibition by serum was less frequent, occurring with 8/71 melanoma patients (6 Stage III, 1 Stage I and 1 Stage II) and no control sera. Preincubation of formalinized tumour cells had no effect on their activity in the leucocyte migration assay, but the preincubation of melanoma or control leucocytes with either autologous serum or foetal calf serum inhibited leucocyte reactivity in approximately 50% of tests. This last effect appears to be immunologically nonspecific.

### SKELETAL METASTASES: RELATIONSHIP OF BONE DESTRUCTION, OSTEOCLAST ACTIVATION AND PROSTAGLANDINS.

C. S. B. Galasko and A. Bennett, *Orthopaedic Unit, Royal Postgraduate Medical School, Hammersmith Hospital, London W12 0HS, and Kings College Hospital, Medical School, Denmark Hill, London SE5 8RX.*

Necropsy and animal experiments indicate that there are two main mechanisms for the bone destruction associated with skeletal metastases. The early, and quantitatively more important mechanism, is mediated *via* osteoclasts, the tumour secreting an osteoclast-activating factor. This is diffusible, but acts only locally in the neighbourhood of the tumour. The second mechanism occurs at a late stage, when the residual trabecula are completely surrounded by tumour cells. At this stage the osteoclasts disappear, although bone destruction continues.

VX2 carcinoma cells were found to synthesize large amounts of prostaglandin-$E_2$-like material, which could be significantly reduced by pretreating the animal with indomethacin ($2{\cdot}4 \pm 0{\cdot}63$ $\mu$g equivalent/g reduced to $0{\cdot}24 \pm 0{\cdot}15$ $\mu$g $PGE_2$ " basal " levels ($P < 0{\cdot}01$); and $4{\cdot}73 \pm 1{\cdot}06$ $\mu$g reduced to $0{\cdot}19 \pm 0{\cdot}07$ $\mu$g " basal + synthesized " levels ($P < 0{\cdot}01$)). Indomethacin pretreatment of rabbits given an intramedullary injection of VX2 carcinoma cell suspension reduced osteoclast proliferation in the neighbourhood of the tumour ($11{\cdot}1 \pm 2{\cdot}13$ osteoclasts reduced to $5{\cdot}2 \pm 1{\cdot}71$ osteoclasts ($P < 0{\cdot}05$), as well as the amount of bone destruction. Since inhibition of osteoclast proliferation and bone destruction were not complete, non-prostaglandin material might also be involved.

The results of these studies suggest that if indomethacin or similar agents are to have a clinical role, they will presumably be useful only in early skeletal metastases associated with osteoclast activation.

**THE EXPRESSION OF THE INTERFERON SYSTEM IN CLONES OF CHINESE HAMSTER/HUMAN HYBRID CELLS.** M. J. MORGAN and P. FAIK, *Department of Biochemistry, University of Leicester, University Road, Leicester LE1 7RH.*

The presence of the interferon system has been investigated in a number of independent hybrid clones derived from a fusion of Chinese hamster cells (CHO-K1; CCl-61) and normal human brain cells. Each clone was characterized on the basis of the electrophoretic mobility of various isozymes, and tested for the production of interferon active in Chinese hamster cells and/or human foreskin fibroblasts, and for the development of virus resistance in response to Chinese hamster interferon and human leukocyte interferon.

The results confirm that human chromosome 21 is required for the anti-virus effect in response to human interferon. Human chromosome 5, but not 2, is essential for human interferon synthesis, and human chromosome 18 may be essential for Chinese hamster interferon synthesis. Only some clones were sensitive to hamster interferon, but it has not been possible to correlate this sensitivity with the presence or absence of a human chromosome.

**ULTRASTRUCTURAL CHANGES AT THE EPITHELIAL–MESENCHYMAL INTERFACE IN EXPERIMENTALLY INDUCED BLADDER TUMOURS.** J. CHOWANIEC and R. M. HICKS, *Department of Histopathology, The Middlesex Hospital Medical School, Riding House Street, London W1P 7LD.*

Ultrastructural alterations were observed at the interface between the neoplastic urothelium and underlying mesenchymal tissues. These were compared to changes induced by cyclophosphamide, which is cytotoxic but not carcinogenic for the urothelium.

Tumours from the bladders of rats subjected to four different experimental treatments were studied by EM. Associated with these tumours, morphological alterations of the basal lamina at the epithelial–mesenchymal interface, and around blood capillaries were noted. These included basal lamina thickening, degeneration or dislocation, and reduplication or lamination. Observations also showed extensive destruction of the underlying collagen, suggesting release of collagenases by the neoplastic urothelium. There was also deposition of fibrin in and around some tumours, possibly due to the inhibition of tissue plasminogen activators. These morphological alterations were associated with progression from *in situ* papillary carcinomas to early, locally invasive urothelial cancers. Human biopsy material from patients with bladder tumours was also studied for comparison.

Similar changes have been documented for other neoplastic tissues, particularly experimentally induced skin tumours in rats, and human oral cancers.

**THE EFFECT OF NICOTINAMIDE ON THE DEVELOPMENT AND LOCALIZATION OF TUMOURS, AND ON THE EXCRETION OF PORPHYRINS IN THE URINE OF RATS GIVEN THE HEPATOCARCINOGEN, DIETHYLNITROSAMINE.** S. GIBBARD and R. SCHOENTAL, *Department of Biochemistry, Princess Alexandra Hospital, Harlow, Middlesex, and Department of Pathology, The Royal Veterinary College, University of London, Royal College Street, London NW1 0TU.*

Pretreatment with nicotinamide can increase the incidence of pancreatic islet-cell tumours in rats given the carcinogens, streptozotocin (Rakieten *et al.* (1971) *Proc. Soc. expt. Biol. Med.*, **137**, 280) and heliotrine (Schoental (1975) *Cancer Res.*, **35**, 2020), known to deplete NAD coenzymes in the liver. Similar treatment with nicotinamide of rats given diethylnitrosamine increased the incidence of kidney tumours; pancreatic islet-cell adenomas were found only rarely.

The increased excretion of urinary porphyrins after diethylnitrosamine was lowered and delayed in rats pretreated with nicotinamide.

**PLASMINOGEN-ACTIVATOR-MEDIATED QUASIMALIGNANT CHARACTERISTICS OF 3T3 CELLS: MORPHOLOGY AND CELL KINETICS.** C. M. URQUHART, E. WRIGHT, P. WHUR, M. GORDON and D. C. WILLIAMS, *Marie Curie Memorial Foundation, Research Department, The Chart, Oxted, Surrey, and City of London Polytechnic.*

3T3 cells co-cultured for 24 h with SV40-3T3 transformants became highly agglutinable by concanavalin A. This induced agglutinability was due to activation of serum plasminogen to plasmin by SV40-3T3 activator. Additional plasmin-mediated effects have now been sought, using this system. Subconfluent monolayers of 3T3 cells in co-culture have altered morphology; the degree of cell spreading was significantly reduced by one-third after 3 days in co-culture, and such cell populations were significantly more trypsin sensitive. However, no change in cell growth kinetics has been detected. The duration of all phases of the cell cycle including G1 was unaltered, and there was no detectable increase in mitotic index in conconfluent cells during the first cycle traverse in co-culture, when compared to controls in monoculture. However, in preliminary experiments we have detected significantly increased levels of [$^3$H]thymidine incorporation into the acid-insoluble fraction of 3T3 cells during the first few cycles in co-culture.

**SURFACE CHANGES IN CHINESE HAMSTER CELLS DURING THEIR PROGRESSION IN CULTURE FOLLOWING CARCINOGEN TREATMENT.** C. J. HARRISON and T. D. ALLEN, *Paterson Laboratories, Christie Hospital and Holt Radium Institute, Manchester M20 9BX.*

Diploid Chinese hamster cells were treated at their first passage with the carcinogens BP, DMBA and MNNG, in DMSO. Controls were treated with the solvent DMSO alone, and the subsequent changes in cell surface morphology were investigated.

The control cells initially had a smooth surface morphology, apart from sparse extremely short microvilli which increased in both length and density with increasing cell passage. Microvillous ridges or " combs " were formed and also increased in frequency as the cells progressed.

The BP-treated cells showed a loss of orientation and began to pile up, 25 passages after treatment. The same trends, of increasing length and microvillous density, and comb formation, with time were observed, as in the controls, but to a much greater extent.

No piling up has yet been observed in either the DMBA- or MNNG-treated cultures. These showed the same characteristics, with time in culture, as above. The DMBA cells maintained a close similarity to the controls in terms of surface morphology, whilst the MNNG cultures were comparable to the BP cells.

It appears therefore that with increasing passage in culture, important surface morphological changes take place, and that these are modified by carcinogen treatment.

**DEPLETED SURFACE SIALO-PEPTIDE IN LEUKAEMIC CELLS.** H. SMYTH and R. O'KENNEDY, *Department of Biochemistry, University College, Belfield, Dublin 4, Eire.*

Surface sialic acids are involved in cellular interactions, immunogenicity and malignancy. Exposure of cells to neuraminidase removes terminal sialic acid in free form, while proteolysis liberates it as a peptide complex. We measured peptide-bound sialic acid released during proteolytic exposure of human peripheral lymphocytes from non-leukaemic and chronic lymphatic leukaemic (CLL) subjects, granulocytes from chronic myeloid leukaemics (CML) and tissue-located lymphocytes from human tonsils and calf thymus. The proteolytic enzyme used was brinase, which enhances human lymphocyte activity *in vivo* (Thornes (1974) *Lancet*, *ii*, 382).

Non-leukaemic peripheral lymphocytes liberated $53{\cdot}5 \pm 6{\cdot}8$ nmol peptide-bound sialic acid/$10^9$ cells (mean $\pm$ s.e., 6 individuals) during mild proteolysis *in vitro*. Values for leukaemic cells were markedly reduced, *viz.*: CLL lymphocytes, $15{\cdot}8 \pm 4{\cdot}2$ (5), $P < 0{\cdot}01$, and CML granulocytes 12·3 (2). Low levels were also noted for non-malignant lymphocytes of tissue origin, that for human tonsillar lymphocytes being $15{\cdot}8 \pm 2{\cdot}0$ (12) and that for calf thymocytes even lower.

The level of neuraminidase-sensitive sialic acid is unaltered in CLL cells (Lichtman and Weed (1970) *Blood*, **35**, 12). Our results show that the protease-sensitive fraction (*ca.*

29%) of this moiety is significantly depleted. Malignant transformation of tissue culture cells is associated with disappearance of a trypsin-sensitive glycoprotein (Hynes (1976) *Biochim. biophys. Acta*, **458**, 73). Our finding may relate to a similar entity in peripheral lymphocytes.

The low levels of protease-sensitive sialic acid in tonsillar lymphocytes and thymocytes may reflect general surface differences between tissue-located lymphocytes and their circulating counterparts.

### STUDIES ON THE MICROCYTOTOXICITY TEST: EVIDENCE THAT THE EFFECTS OF NORMAL LYMPHOID CELLS ON THE GROWTH AND SURVIVAL OF SYNGENEIC TUMOUR CELLS IN MICROTEST PLATES ARE CAUSED BY NON-IMMUNOLOGICAL MODIFICATIONS OF THE CULTURE MEDIUM.

R. C. Rees and C. G. Brooks, *Cancer Research Campaign Laboratories, University of Nottingham.*

Despite many years of intensive research, it is still not clear whether lymphoid cells from human cancer patients possess any specific cytotoxic activity towards the hosts' tumour cells, similar to that demonstrated in various animal systems. The major problem has been the high, but variable, "non-specific" cytotoxic activity of leucocytes obtained from control patients. To try to elucidate the mechanisms of this "non-specific" killing, we have studied what appears to be a similar phenomenon in inbred rats. Thus, lymph node cells (LNC) from *normal* Wistar rats were found either to inhibit or to enhance the growth and survival of syngeneic solid tumour cells cultured in micro-test plates. Which effect was observed depended on the particular tumour cell used and, in some cases, on the particular *in vitro* subline used. The cells responsible for inhibition were selectively retained on nylon-wool columns, whereas the cells responsible for enhancement were selectively eluted. When these cell fractions were incubated in the absence of tumour cells, the cell-free supernatants mediated the same effects as the cells from which they were derived. The supernatant inhibitory activity was maximal within 1 h of cell culture, whereas the supernatant enhancing activity developed over a 48-h period. Furthermore, tumour cells themselves produced growth-enhancing activity during culture in microplates. It is proposed that interaction between these various supernatants is responsible, at least in part, for the non-specific effects of normal lymphoid cells in the microcytotoxicity test.

### IMMUNOLOGICAL STUDIES OF PATIENTS WITH PRIMARY BREAST CARCINOMA.

R. A. Robins, C. J. Davies, I. D. Meredith, R. W. Blamey and R. W. Baldwin, *Cancer Research Campaign Laboratories and Department of Surgery, The University, Nottingham NG7 2RD.*

Immunological tests with primary breast carcinoma patients were undertaken, to evaluate their usefulness in prognosis of individual patients. General cellular immunocompetence was tested by response to dinitrochlorobenzene (DNCB); sensitization was detected both by skin testing and *in vitro* leucocyte migration inhibition to DNCB. "Specific" cellular reactivity to an extract of pooled breast carcinoma tissue was measured in a leucocyte migration inhibition test. Results of the immunological tests were compared with prognosis determined by the triple lymph node biopsy (TNB) method of Handley. Preliminary survival data indicate that this method is giving reliable results in our series of patients.

The ability to respond to DNCB, whether detected by *in vitro* test or skin test, did not correlate with prognosis determined by TNB; indeed the best correlate of DNCB unresponsiveness was patients' age.

Responses to breast carcinoma extract were detected, using breast carcinoma patients' leucocytes at concentrations giving low reactivity in controls. No correlation between reactivity and TNB prognosis has so far been observed.

### K-CASEIN CONCENTRATIONS IN PATIENTS WITH BENIGN AND MALIGNANT BREAST DISEASES.

R. L. Woods, F. S. Searle and K. D. Bagshawe, *Department of Medical Oncology, Charing Cross Hospital, Fulham Palace Road, London W6 8RF.*

A radioimmunoassay was set up for the breast protein K-casein. Of 361 samples from healthy volunteers and from patients with non-malignant diseases, only 7 gave values greater than 60 ng/ml. Sixty-two samples from lactating women gave values ranging from 136 ng/ml to 1700 ng/ml.

A number of samples from patients with breast diseases were assayed: 57% of patients with metastatic breast cancer had concentrations more than 60 ng/ml, whilst 39% of patients with cancer confined to the breast and its regional nodes had elevated values. Of those patients with treated breast cancer but clinically tumour-free at the time of assay, 17% had elevated concentrations. Nine per cent of patients with histologically proven benign breast disease had values greater than 60 ng/ml.

No correlation was found between age, endocrine status or histological type and casein levels.

A radioimmunoassay for α-lactalbumin showed elevated concentrations in only 1/70 samples assayed from patients with proven carcinoma of the breast.

**CYTOTOXICITY OF SUBPOPULATIONS OF IMMUNE LYMPHOID CELLS, AND ANTIBODY-DEPENDENT CELLULAR CYTOTOXICITY AGAINST GUINEA-PIG HEPATOMA CELLS *IN VITRO*.** M. ANDJARGHOLI and M. M. DALE, *Department of Pathobiology, Institute of Public Health Research, University of Teheran, P.O. Box 1310, Teheran, Iran, and Department of Pharmacology, University College, London WC1.*

A microassay was used for measuring the cytotoxicity of guinea-pig lymphoid cells against allogeneic tumour cells in culture.

The activity of subpopulations of immune lymphocytes was investigated. From the results obtained, it appeared that although both T-cell-enriched and B-cell-enriched cell populations exhibit cytotoxicity in our tumour system, the crude cell population is the most effective of all. This probably suggests that there is cell cooperation in producing cytotoxicity in this system.

Antibody-dependent cellular cytotoxicity was also investigated in this system. It was found that, under optimum conditions, this type of cytotoxicity could be obtained concomitantly with direct cell-mediated cytotoxicity. A B-cell-enriched population of normal cells was shown to become cytotoxic to tumour cells in the presence of a high dilution of serum from immune animals.

**THE IDENTIFICATION OF INFILTRATING HOST CELL TYPES IN RAT TUMOURS OF VARYING IMMUNOGENICITY.** K. MOORE and M. MOORE, *Paterson Laboratories, Christie Hospital and Holt Radium Institute, Manchester M20 9BX.*

The numbers of host infiltrating cells found in enzyme-disaggregated rat tumours has been measured by the formation of erythrocyte-antibody (EA) rosettes to detect Fc-receptor-bearing cells, erythrocyte antibody complement (EAC) rosettes to detect cells bearing 3C receptors, and macrophages identified by their ability to phagocytose bound EA and polystyrene particles. Other cell types were identified morphologically in stained cytocentrifuge preparations.

Serially transplanted tumours of high immunogenicity (MC40A, MC57) were found to contain a greater proportion of macrophages (12·2 ± 0·5% and 15·2 ± 1·7% respectively) than those of low immunogenicity (SP1, SP22, AAF57: 9·7 ± 1·4%, 8·5 ± 2·2% and 4·7 ± 1·1% respectively). Also the immunogenic tumours contained relatively high numbers of non-phagocytic EA rosette-forming cells (14·1% and 13·9%) compared with the negligible numbers found in the tumours of low immunogenicity (2·4%, 3·1% and 0·3%). None of the tumours contained more than 2·5% of EAC rosette-forming cells, polymorphonuclear leucocytes, or basophils.

The kinetics of infiltration of tumours derived from an inoculum of $10^6$ tumour cells which had been depleted of host cells by a number of *in vitro* passages were measured. Initially, an increase in the number of macrophages relative to that found in the transplanted tumours was detected in immunogenic and non-immunogenic tumours, followed by a decline to the levels found in the transplanted tumours. A marked difference was the total absence of non-phagocytic EA rosette-forming cells in one non-immunogenic tumour (SP22) throughout the time course, compared with the increase of these cells with time in the immunogenic tumour (MC40A).

**RESPONSE OF ASTROCYTOMA CULTURES TO THERAPEUTIC CONCENTRATIONS OF DEXAMETHASONE AND BETAMETHASONE.** M. GUNER, R. I. FRESHNEY, D. MORGAN, M. G. FRESHNEY and D. G. T. THOMAS, *Beatson Institute and Institute of Neuroscience, Glasgow.*

The use of steroids in the management of patients with brain tumours has raised the question whether these drugs may be cytotoxic to the tumour cells. This has been examined at the cellular level in cultures prepared by collagenase digestion of biopsy specimens of 1 intermediate and 4 anaplastic astrocytomas. Secondary cultures were shown to be predominantly aneuploid (hypodiploid) and proliferative (88–97% labelling index with [$^3$H]thymidine). They were trypsinized, and clonal growth was measured in the presence and absence of different concentrations of dexamethasone and betamethasone. The cloning efficiencies of control cultures varied from 2·8 to 10·6% in Ham's F12 medium supplemented with Eagle's MEM amino acids and 20% foetal bovine serum, buffered in 20 mM Hepes and with no additional $CO_2$ in the gas phase. Stimulation of cloning efficiency was obtained in every case (5 cell lines, 6 experiments) reaching a maximum at 12·5 $\mu$g/ml with both steroids. The percentage stimulation varied from 50% to 250%, and toxicity was only produced at 50 $\mu$g/ml in most cases. Dexamethasone was generally found to be more toxic than betamethasone. Analysis of colony size (cells/colony) showed a similar pattern, with maximum stimulation of colony size at 12·5 $\mu$g/ml of either steroid. Similar clonal cultures from an acromegalic pituitary tumour showed 2·3 times higher cloning efficiency in dexamethasone, but reduced colony size at 12·5 $\mu$g/ml. A similar effect was also found with normal diploid fibroblasts. So far, only astrocytoma cultures have shown increased proliferation in dexamethasone or betamethasone, and only in conditions of low $CO_2$ and bicarbonate concentration.

Under these particular conditions, dexamethasone and betamethasone promote clonal growth in astrocytoma cultures, and are found to be cytotoxic only at concentrations unlikely to be found *in vivo*. It is now intended to test the effects of these steroids at higher cell densities, and *in vivo*, to determine the potential clinical relevance of these findings.